# Binding Sites in the *EFG1* Promoter for Transcription Factors in a Proposed Regulatory Network: A Functional Analysis in the White and Opaque Phases of *Candida albicans*

**DOI:** 10.1534/g3.116.029785

**Published:** 2016-04-20

**Authors:** Claude Pujol, Thyagarajan Srikantha, Yang-Nim Park, Karla J. Daniels, David R. Soll

**Affiliations:** Developmental Studies Hybridoma Bank, Department of Biology, University of Iowa, Iowa 52242

**Keywords:** *cis*-acting sequences, white-opaque transition, differential gene expression

## Abstract

In *Candida albicans* the transcription factor Efg1, which is differentially expressed in the white phase of the white-opaque transition, is essential for expression of the white phenotype. It is one of six transcription factors included in a proposed interactive transcription network regulating white-opaque switching and maintenance of the alternative phenotypes. Ten sites were identified in the *EFG1* promoter that differentially bind one or more of the network transcription factors in the white and/or opaque phase. To explore the functionality of these binding sites in the differential expression of *EFG1*, we generated targeted deletions of each of the 10 binding sites, combinatorial deletions, and regional deletions using a *Renilla* *reniformis* luciferase reporter system. Individually targeted deletion of only four of the 10 sites had minor effects consistent with differential expression of *EFG1*, and only in the opaque phase. Alternative explanations are considered.

Chromatin immunoprecipitation followed by genome-wide chip hybridization (ChIP-chip), provides a tool for identifying transcription factor (TF) binding sites in the upstream regulatory regions of genes that are differentially expressed in alternative phenotypes or under different environmental conditions (Sun *et al.* 2010; Qin *et al.* 2011; Vernes *et al.* 2011; Yu *et al.* 2011; Cho *et al.* 2012; Kwon *et al.* 2012; Federowicz *et al.* 2014). By combining ChIP-chip hybridization analyses with mutational analyses and genome-wide transcription profiling, transcriptional networks regulating phenotypic transitions and the expression of alternative phenotypes can be developed (Sun *et al.* 2010; Qin *et al.* 2011; Vernes *et al.* 2011; Wang *et al.* 2011; Cho *et al.* 2012; Kwon *et al.* 2012; Federowicz *et al.* 2014). However, while ChIP-chip analyses provide the locations of binding sites, they do not assess functionality (Anderson *et al.* 1989; Li *et al.* 2008; Cooke *et al.* 2009; Ucar *et al.* 2009; Qin *et al.* 2011; Carey *et al.* 2012; Maienschein-Cline *et al.* 2012; Whitfield *et al.* 2012; Nguyen-Duc *et al.* 2013; Teytelman *et al.* 2013; Wei *et al.* 2013; Wu and Ji 2013; Cusanovich *et al.* 2014; DeVilbiss *et al.* 2014; Sanalkumar *et al.* 2014; Slattery *et al.* 2014; Bansal *et al.* 2015). The effects of deleting or perturbing expression of a TF gene can help identify TF-binding site interactions that may be nonfunctional or for which there is redundancy, but they do not demonstrate functionality, since the effects may be indirect (Cooke *et al.* 2009; Qin *et al.* 2011; Maienschein-Cline *et al.* 2012; Wei *et al.* 2013; Wu and Ji 2013). Although there is no single “gold standard” method for unequivocally testing the functionality of TF-binding site interactions (Carey *et al.* 2012; Slattery *et al.* 2014), one relatively effective method is through the construction and analysis of deletion derivatives of the binding sites, using quantitative reporter gene strategies (Mönke *et al.* 2012; Whitfield *et al.* 2012; Slattery *et al.* 2014; Lin *et al.* 2015; Suzuki *et al.* 2015; Taka *et al.* 2015). Here, we have used this strategy to explore the functionality of TF binding sites in the upstream region of *EFG1* (orf19.610 ) in *Candida albicans*. *EFG1* (orf19.610 ) encodes a MyoD-class helix-loop-helix TF (Berkes and Tapscott 2005; Hu *et al.* 2008) that is differentially expressed in the white and opaque phases of *MTL*-homozygous cells, is essential for expression of the white phenotype, and negatively regulates the transition from the white to opaque phase (Sonneborn *et al.* 1999; Srikantha *et al.* 2000; Lachke *et al.* 2003; Zordan *et al.* 2007).

*C. albicans*, the most pervasive yeast pathogen colonizing humans (Odds 1988, 1998; Hobson 2003; Pfaller and Diekema 2007; Huffnagle and Noverr 2013; McManus and Coleman 2014), is diploid and natural isolates are predominately heterozygous (**a**/α) at the mating type locus (*MTL*) (Lockhart *et al.* 2002; Rustad *et al.* 2002; Legrand *et al.* 2004; Odds *et al.* 2007; Basma *et al.* 2009). To mate, **a**/α cells must undergo homozygosis to the **a/a** or α/α configuration (Hull and Johnson 1999; Hull *et al.* 2000; Magee and Magee 2000), then switch (Slutsky *et al.* 1987) from the “white” to “opaque” phase (Lockhart *et al.* 2002, 2003; Miller and Johnson 2002). The capacity to undergo white-opaque switching is also required for the formation of a *MTL*-homozygous white cell “sexual” biofilm, which facilitates mating between opaque cells (Daniels *et al.* 2006; Yi *et al.* 2008, 2011a,b; Park *et al.* 2013; Soll 2014).

In 1999, Sonneborn *et al.* (1999) found that *EFG1* (orf19.610 ) played a role in regulating the opaque to white transition. *MTL*-homozygous deletion mutants of *EFG1* (orf19.610 ) were blocked in the opaque phase. In the following year, Srikantha *et al.* (2000) demonstrated that cells of the *EFG1* (orf19.610 ) null mutant attempted to switch from opaque to white when the temperature was raised, but could not fully express the white cell phenotype. In 2006, three laboratories reported that *WOR1* (orf19.4884 ) regulated the white to opaque transition (Huang *et al.* 2006; Srikantha *et al.* 2006; Zordan *et al.* 2006). *MTL*-homozygous deletion mutants of *WOR1* (orf19.4884 ) were blocked in the white phase. Soon after the discovery of Wor1, Zordan *et al.* (2007) used a combination of double mutants, ectopic expression, and ChIP-chip analyses to develop a model of a transcriptional network of interacting TFs that regulated the white and opaque phenotypes. The interacting network included the TFs Efg1, Wor1, Wor2, and Czf1 (Zordan *et al.* 2007). The network was then expanded to include the TFs Ahr1 (Wang *et al.* 2011) and Wor3 (Lohse *et al.* 2013). Hernday *et al.* (2013) then performed an in depth analysis of the six TFs, including genome-wide ChIP-chip analysis, gene expression profiling, and microfluidics-based DNA binding studies. Transcriptional models were then generated for the expression of the alternative phenotypes, each model based in TF binding to sites along the promoters of the six TFs. Based on the phenotypes of the deletion mutants, there appeared to be a hierarchy in the roles played by components of the networks. If *WOR1* (orf19.4884 ) was deleted, cells were blocked in the white phase (Huang *et al.* 2006; Srikantha *et al.* 2006; Zordan *et al.* 2006) and if *EFG1* (orf19.610 ) was deleted, cells were blocked in the opaque phase (Sonneborn *et al.* 1999; Srikantha *et al.* 2000). However, deletion of *AHR1* (orf19.7381 ) resulted in a decrease in the frequency of switching from opaque to white (Wang *et al.* 2011), deletion of *WOR2* (orf19.5992 ) or *CZF1* (orf19.3127 ) resulted in a decrease in the frequency of switching from white to opaque (Vinces and Kumamoto 2007; Zordan *et al.* 2007), and deletion of *WOR3* (orf19.467 ) had no effect on switching (Lohse *et al.* 2013). In addition, deletion of *WOR2* (orf19.5992 ) had no effect on N-acetylglucosamine-induced switching from white to opaque (Tong *et al.* 2014). Overexpressing *CZF1* (orf19.3127 ) or *WOR3* (orf19.467 ) caused an increase in the frequency of switching from white to opaque, and both increases were dependent on *WOR1* (orf19.4884 ) (Vinces and Kumamoto 2007; Lohse *et al.* 2013; Xu *et al.* 2015). Overexpression of *AHR1* (orf19.7381 ) caused an increase in the frequency of opaque to white switching, and the increase in this case was dependent on *EFG1* (orf19.610 ) (Wang *et al.* 2011). Overexpression of *WOR2* (orf19.5992 ) had no effect on switching (Zordan *et al.* 2007). Together, these observations supported a model in which *EFG1* (orf19.610 ) was the major regulator of the white phase phenotype and *WOR1* (orf19.4884 ) the major regulator of the opaque phase phenotype, while the remaining components of the network functioned as modulators of *EFG1* (orf19.610 ) and *WOR1* (orf19.4884 ) expression.

*EFG1* (orf19.610 ) is differentially expressed in white phase cells at levels over 10 fold higher than in opaque phase cells (Sonneborn *et al.* 1999; Srikantha *et al.* 2000; Lachke *et al.* 2003; Zordan *et al.* 2006). We previously demonstrated different transcription start points (TSPs) for *EFG1* (orf19.610 ) in the two phases, resulting in a 3.3 kb transcript in the white phase and a 2.1 kb transcript in the opaque phase (Srikantha *et al.* 2000). Hernday *et al.* (2013) demonstrated that there are a total of 10 binding sites for various combinations of the network TFs in a 10 kb region immediately upstream of the *EFG1* (orf19.610 ) open reading frame (ORF) (Figure 1A). In the white phase, in which *EFG1* (orf19.610 ) expression is high, four of these sites bind only Efg1 (sites 1, 2, 3, and 6), two only Ahr1 (sites 7 and 8), and one Efg1 and Czf1 (site 9) (Figure 1A). In the opaque phase, in which *EFG1* (orf19.610 ) expression is low, two of the 10 sites bind Ahr1 (sites 5 and 8), one Wor2 and Efg1 (site 1), one Wor1, Wor2, and Wor3 (site 10), one Wor1, Wor2, Czf1, and Efg1 (site 9), and two Wor1, Wor2, Wor3, Czf1, and Efg1 (sites 4 and 6) (Figure 1A). Of the 10 binding sites, site 10 is located between the white and opaque TSPs, and therefore resides in the region encoding the nontranslated portion of the white phase *EFG1* (orf19.610 ) transcript.

**Figure 1:**
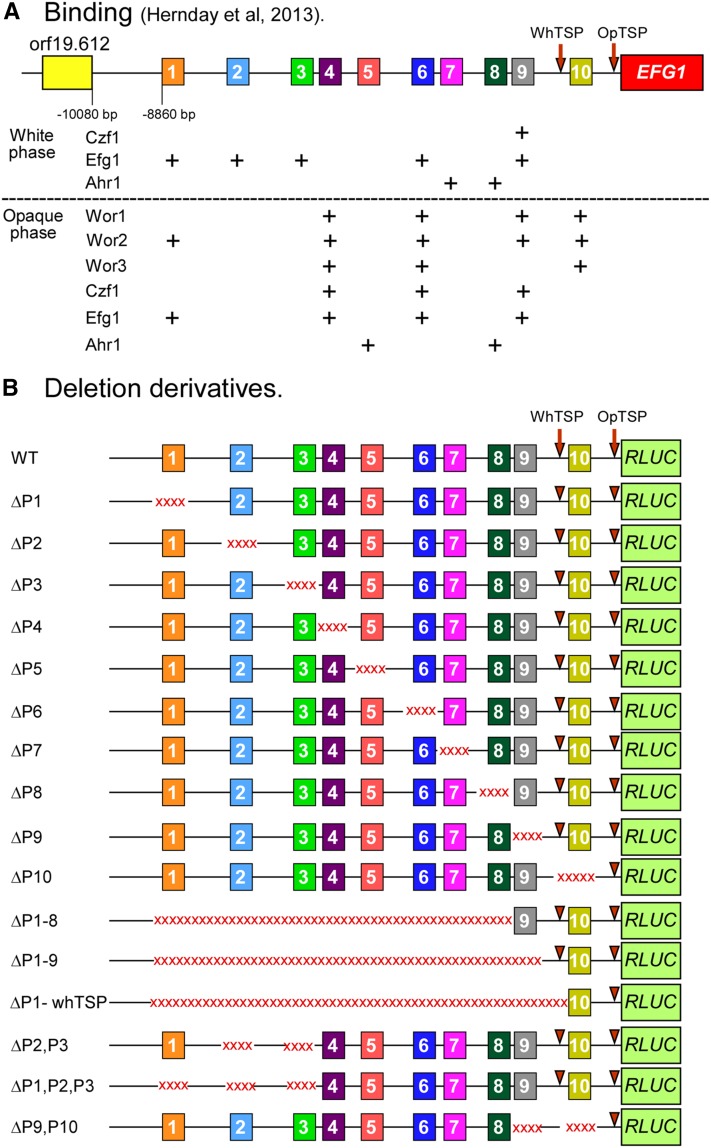
The 10 binding sites of network transcription factors regulating *EFG1* and the deletion derivatives generated for this functional analysis in the α/α strain WO-1. (A) Binding of transcription factors to the 10 sites in the *EFG1* upstream region, based on the ChIP-chip data of Hernday *et al.* (2013). Orf19.612 is the first upstream gene to *EFG1*. (B) A description of the deletion derivatives generated. Deleted regions are shown as red x sequences. ChIP-chip, chromatin immunoprecipitation followed by genome-wide chip hybridization; OpTSP, opaque transcription start point; RLUC, *Renilla reniformis* luciferase; WhTSP, white transcription start point.

Prior to the discovery of the network TF binding sites along the *EFG1* (orf19.610 ) promoter (Hernday *et al.* 2013), we generated a series of 22 serial deletion derivatives beginning at –2320 bp and progressing sequentially to the *EFG1* (orf19.610 ) ORF (Lachke *et al.* 2003), using the *Renilla reniformis* gene RLUC as a quantifiable transcription reporter (Srikantha *et al.* 1996). However, that study (Lachke *et al.* 2003) was limited for several reasons. First, the promoter region analyzed included only binding sites 9 and 10. Second, the deletion derivatives of the limited promoter fused to *RLUC* were inserted downstream of a full length *EFG1* (orf19.610 ) allele (Supplemental Material, Figure S1). We have therefore performed a second functional analysis of the *EFG1* (orf19.610 ) promoter, using a different strategy, in order to explore the role of the 10 binding sites in the differential expression of *EFG1* (orf19.610 ) in the white and opaque phases. Individual deletion derivatives were generated for each of the 10 binding sites, as well as combinatorial deletions, in a region spanning the 9000 bp upstream region of *EFG1* (orf19.610 ). These deletion derivatives of the promoter were generated upstream of *RLUC*, which replaces the *EFG1* (orf19.610 ) ORF in one of the *EFG1* (orf19.610 ) alleles, in the α/α strain WO-1 (Slutsky *et al.* 1987) and the **a/a** strain P37005 (Lockhart *et al.* 2002), a strategy very different to the previous one (Figure S1). We show that individual deletion of any one of the nine binding sites upstream of the white phase transcription start point (WhTSP) of *EFG1* (orf19.610 ) has no significant negative effect on the elevated level of reporter gene expression in the white phase. Individual deletion of sites 1, 2, 3, and 4 resulted in minor increases in RLUC activity in the opaque phase, but deletion of sites 5, 6, 7, 8, and 9 did not. Combinatorial deletion of binding sites 1, 2, and 3 showed no negative effects in white phase activity and no additive effect of the minor increases in activity in the opaque phase. Full length deletions of the upstream region, including sites 1 through 8, 1 through 9, or 1 through the WhTSP, resulted in a decrease in white phase expression to the low level observed in opaque phase cells. These full length deletions have no positive effect on the low level of expression in the opaque phase. They actually cause a further decrease in the already low level. These results indicate, first, that the high level of *EFG1* (orf19.610 ) expression in the white phase is dependent upon the upstream region, but our strategy provided no support for the idea that any one of seven binding sites in the white phase plays an essential and nonredundant role in the increased level of *EFG1* (orf19.610 ) expression in that phase. These results suggested that sites 1, 2, 3, and 4 may play minor suppressive roles in the opaque phase, but those roles were not additive. Interpreting these results is not straightforward, given that there is no single gold standard for definitively assessing the functionality of *cis*-acting sequences that bind TFs in the regulation of alternative phenotypes (Ucar *et al.* 2009; Carey *et al.* 2012; Spivakov 2014). Alternative interpretations are considered in the *Discussion* to explain these results.

## Material and Methods

### Yeast strains and growth conditions

The mutant strains used in this study were derived from two clinical *C. albicans* strains, WO-1 (Slutsky *et al.* 1987), the *MTL* configuration of which is α/α, and P37005 (Lockhart *et al.* 2002), the *MTL* configuration of which is **a/a**. Strains and their deletion derivatives are described in Table S1. Cells were grown from stocks stored at −80° and maintained at 25° on agar plates containing Lee’s medium (Lee *et al.* 1975) supplemented with arginine, biotin, and zinc (sLee’s medium) (Bedell and Soll 1979), and containing 5 µg/ml phloxine B, which differentially stains opaque colonies and sectors red (Anderson and Soll 1987). The phenotypes of cells in white and opaque preparations were verified microscopically to be at least 99% homogeneous prior to use in every experiment.

### Construction of R. reniformis luciferase (RLUC) reporter strains

The strategy is diagrammed in Figure S2. The *EFG1* (orf19.610 ) ORF in one of the two alleles was disrupted by inserting *RLUC*, in strains WO-1 and P37005, to create the wild type (“WT”) reporter strains by the following procedure. The fragment *EFG1*-5′, containing the 853 bp upstream of the ATG codon of the *EFG1* (orf19.610 ) ORF, was amplified by polymerase chain reaction (PCR) with the primers efg5primef1 and efg5primer1.2 using WO-1 DNA as template. All primers used in this study are described in Table S2. The fragment EFG1-3ˈ, containing a portion of the *EFG1* (orf19.610 ) ORF (base pairs 179–926), was amplified with the primers efg3primef2-2 and efg3primer2. The fragments *EFG1*-5′ and *EFG1*-3′ were digested with *Pst*I and ligated. The 5′–3′ fusion product was digested with *Sma*I and inserted between the flanking sites of the *Sma*I-digested, dephosphorylated pGEM-7Z(f) vector (Promega) to create plasmid pEf5/3-5.1. The *C. albicans* adapted hygromycin B resistance gene (*HYG^R^*) was amplified with primers CaHygB-1 and CaHygB-2 from plasmid pBSH-CaHygB (Basso *et al.* 2010) and digested with *Sal*I and *Nde*I. The *ACT1* promoter was amplified with primers ACT1P-1 and ACT1P-2 from plasmid pNIMI (Park and Morschhäuser 2005) and digested with *Xho*I. The *ACT1* terminator sequence was excised from pNIMI by digestion with *Nde*I and *Pst*I. The *ACT1* promoter, *HYG^R^*, and the *ACT1* terminator were cloned in the pBluescript II SK+ (Stratagene) plasmid, between *Xho*I and *Pst*I sites of this vector to create plasmid CaHygB. The 1.7 kb fragment containing the *Escherichia coli* hygromycin-resistance gene under the control of the *ACT1* promoter and terminating with the *ACT1* terminator sequence (Figure S1), was amplified from plasmid pCaHygB with the primers hynancof-2a and hynaw3ncor-2, digested with *Bsp*hI, and inserted into the plasmid pCRW3 (Srikantha *et al.* 1996) at the compatible *Nco*I site, located downstream of the *RLUC* gene, to create plasmid pCRH8. The 3.2 kb fragment containing *RLUC* and the hygromycin resistance gene was amplified from plasmid pCRH8 with the primers rlucsbff-2 and hynaw3ncor-2, digested with *Pst*I, and subcloned into plasmid pEf5/3-5.1 at the *Pst*I site, located at the junction between the *EFG1*-5′ and *EFG1*-3′ fragments, to create plasmid p6bW3-8. The orientation of the *RLUC* gene was verified by PCR and sequencing. p6bW3-8 was digested by *Sma*I to generate the *RLUC* cassette (Figure S1). The *RLUC* cassette was used to transform strains WO-1 and P37005 by electroporation (De Backer *et al.* 1999). Transformants were selected on YPD (1% yeast extract, 2% peptone, and 2% dextrose) agar containing 800 µg/ml of hygromycin B (InvivoGen) after 2 d of growth at 30°. Correct integration of the *RLUC* cassette in one of the *EFG1* (orf19.610 ) alleles was verified by PCR with the primers efg5′chk and rlucrchk2, and by sequencing. Several clones containing the *RLUC* cassette at one of the two *EFG1* (orf19.610 ) alleles were obtained in each strain background. Four clones derived from WO-1 and four clones derived from P37005 were tested for RLUC activity in white and opaque phase cells. Strain F1, derived from WO-1, was selected for generating deletion derivatives of the *EFG1* (orf19.610 ) promoter regulating *RLUC*, and strain I1 was selected for P37005. We considered those strains, F1 and I1, in which *RLUC* is under the control of the complete 10,800 bp upstream region of *EFG1* (orf19.610 ), to be the “wild type” (“WT”) strains.

### Construction of EFG1 **(**orf19.610 **)** promoter deletion derivatives

The deletion mutants, listed in Table S1 and diagrammed in Figure 1B, were generated according to previously described protocols (Yi *et al.* 2008; Srikantha *et al.* 2012, 2013). The recyclable flipper cassette from pGEM2A (Reuß *et al.* 2004; Srikantha *et al.* 2012), containing the dominant nourseothricin resistance marker (*CaSAT1*), was used to create all mutants. The primers used to create gene deletions are provided in Table S2. To obtain mutants with individual binding site deletions, combinations of targeted deletions, or major contiguous deletions of the upstream region, specific deletion cassettes were constructed as follows. First, the 5′- and 3′-flanking regions of each target region were amplified by PCR. The 5′ and 3′ regions were then digested by *Sma*I and ligated together. The 5′–3′ fusion product was amplified by PCR and subcloned into the pGEM-T Easy vector (Promega). The *Sma*I-digested *SAT*-flipper cassette from pGEM2A (Srikantha *et al.* 2012) was then inserted between the flanking fragments of the *Sma*I-digested, dephosphorylated plasmid. The resulting plasmids were digested with *Not*I to generate each deletion cassette used to transform the F1 or I1 *RLUC* reporter strains by electroporation (De Backer *et al.* 1999). Transformants were selected on YPD agar containing 200 µg/ml nourseothricin (ClonNAT, WERNER BioAgents) after 3 d of growth at 30°. Transformants were then assessed, by PCR, for the correct insertion of the deletion cassette in the *EFG1* (orf19.610 ) promoter allele controlling the *RLUC* gene. Two or more independent clones were obtained for each deletion derivative. These were then subjected to a pop-out strategy in the maltose-containing medium YPM (1% yeast extract, 2% peptone, and 2% maltose), to excise the *CaSAT1* marker. Multiple site mutations were performed by repeating the transformation and selection processes.

### In vitro luciferase assay

Luciferase activity was assayed according to methods previously described in detail (Srikantha *et al.* 1996; Lachke *et al.* 2003), with minor modifications. White and opaque cells were grown to late log phase in modified Lee’s liquid medium for 20 hr at 30°. Cells were washed once with sterile distilled water and once with RLUC buffer [0.5 M NaCl, 0.1 M K_2_HPO_4_ (pH 6.7), 1 mM EDTA, 0.6 mM sodium azide, 1 mM phenylmethylsulphonyl fluoride (PMSF), 0.02% BSA] without BSA. Two volumes of zirconia/silica beads (0.5 mm diameter, Research Product International Corp.) were added to the cell pellet in 200 µl of RLUC buffer without BSA. Cells were disrupted with a BeadBeater (BioSpect Products) and centrifuged at 13,000 × *g* for 10 min at 4°. Five µl of the cell-free extract were added to 100 µl of RLUC buffer, without sodium azide or PMSF, supplemented with 1 µM coelenterazine (Molecular Probes, Inc.). The RLUC buffer used in the assay mixture was flushed with nitrogen gas for 4 min to prevent auto-oxidation of the coelenterazine. Immediately after mixing in a Luminometer Cuvette (BD Monolight, BD Biosciences), light emission was measured at 480 nm in the integration mode for 30 sec with a Monolight 2010 luminometer (Analytical Luminescence Laboratory). RLUC activity was measured in relative light units, defined as light emitted per 30 sec per µg of protein. Protein concentrations were estimated using the Micro BCA Protein Assay Kit (Thermo Scientific). The luciferase activity data presented for each promoter mutant represent the means of at least three independent experiments obtained for two independent clones. Strains are available upon request.

### Data availability

The authors state that all data necessary for confirming the conclusions presented in the article are represented fully within the article.

## Results

### Strategy

In our previous analysis of the *EFG1* (orf19.610 ) promoter, we employed a strategy in which the region upstream of the *EFG1* (orf19.610 ) ORF was serially deleted, each deletion beginning at base pair –2320 (Lachke *et al.* 2003), which proved to represent only a fourth of the full upstream region of *EFG1* (orf19.610 ). The deletion derivatives of the *EFG1* (orf19.610 ) promoter were placed upstream of the *Renilla* luciferase gene *RLUC*, and the constructs inserted downstream of either one of the two *EFG1* (orf19.610 ) alleles (Figure S1). Here, we have taken a different strategy that involves the entire upstream region of *EFG1* (orf19.610 ), including sites 1 through 10, does not include the wild-type promoter and ORF of *EFG1* (orf19.610 ) upstream of the reporter construct, and is restricted to the same *EFG1* (orf19.610 ) allele (Figure S1). The deletion derivatives targeted individual network TF binding sites, identified in the ChIP-chip analysis of Hernday *et al.* (2013). We generated two independent deletion derivatives in the α/α strain WO-1 for each individual binding site, for combinations of individual binding sites, for sites 1, 2, and 3, and for sites 9 and 10, and contiguous deletions from –8860 bp through site 8, from −8860 bp through site 9, and from −8860 bp through the WhTSP, as diagrammed in Figure 1B and described in Table S3. Select deletion derivatives of individual binding sites and contiguous deletion from −8860 bp through site 9 were also generated for **a/a** strain P37005. For the derivatives generated in strains WO-1 (α/α) and P37005 (**a/a**), RLUC activity was assessed for the two independent strains of each deletion derivative in the white and opaque phase. Each deletion derivative was assessed in triplicate and the data of the independent deletion derivatives pooled.

### Differential expression of RLUC in the white phase

**Figure 2:**
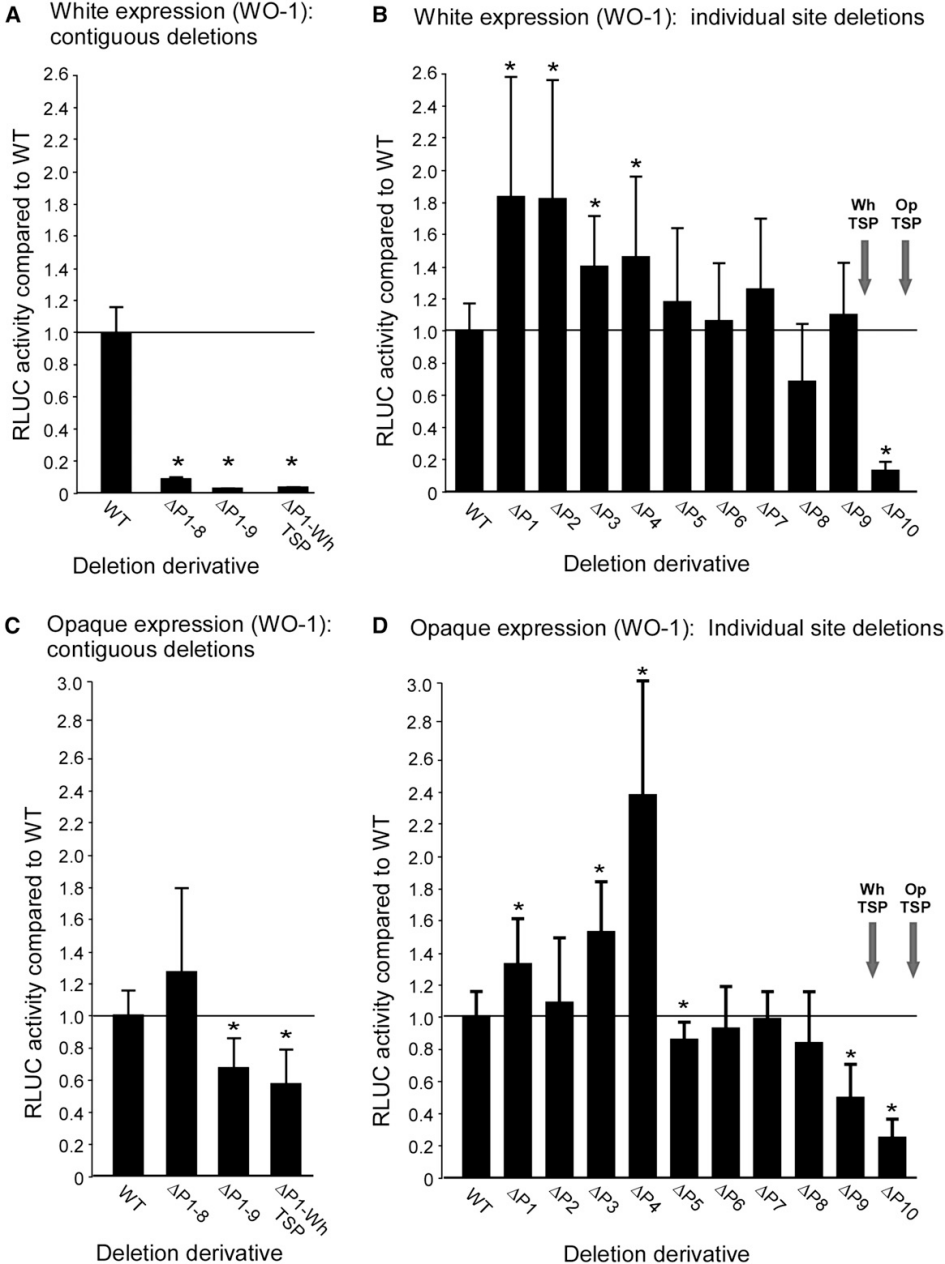
RLUC activity of deletion derivatives generated in the α/α strain WO-1, compared to the wild type (“WT”) derivative containing the full length upstream region *EFG1*. RLUC activities are plotted in relation to that of the WT activity in the same phase, which was set at 1.0. (A) RLUC activity in the white phase of contiguous deletions from −8860 bp through binding site 8 (ΔP1-8), binding site 9 (ΔP1-9), and the WhTSP. (B) RLUC activity in the white phase of targeted deletions of the 10 transcription factor binding sites. (C) RLUC activity in the opaque phase of contiguous deletions from −8860 bp through binding site 8, binding site 9, and the WhTSP. (D) RLUC activity in the opaque phase of targeted deletions of the 10 transcription factor binding sites. Error bars represent standard deviation. Starred bars indicate a significant difference with WT. (p < 0.05; two-tailed Mann–Whitney nonparametric test). OpTSP, opaque transcription start point; RLUC, *Renilla reniformis* luciferase; WhTSP, white transcription start point.

**Figure 3:**
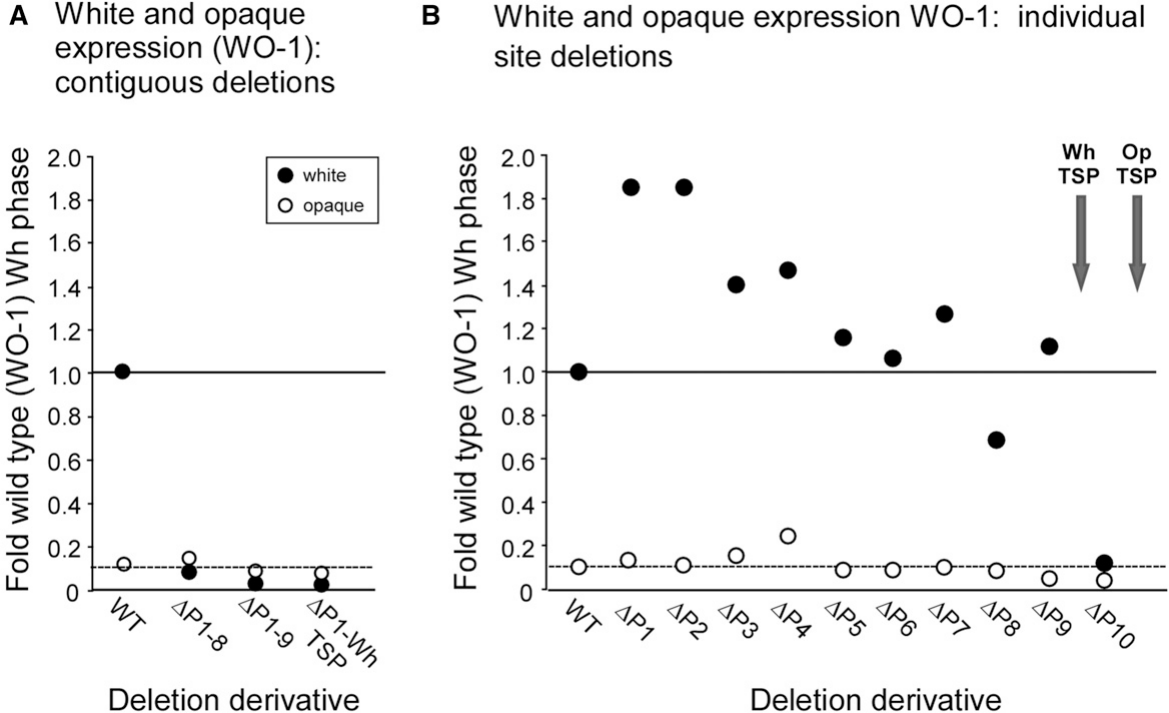
RLUC activity of deletion derivatives generated in α/α strain WO-1, in either the white or opaque phase, relative to activity of the WT derivative in the white phase. (A) RLUC activity of contiguous deletions. (B) RLUC activity of the targeted deletions of the 10 transcription factor binding sites. OpTSP, opaque transcription start point; RLUC, *Renilla reniformis* luciferase; WhTSP, white transcription start point; WT, wild type.

Previously, northern blot analysis demonstrated that *EFG1* (orf19.610 ) of strain WO-1 (α/α) was differentially expressed at levels approximately 10 × higher in the white phase than in the opaque phase, and that the transcript was larger in the white phase (3.2 kb) than in the opaque phase (2.2 kb), a result of different TSPs (Sonneborn *et al.* 1999; Srikantha *et al.* 2000). RLUC activity of the full length promoter region (WT) in the WO-1 α/α strain (Figure 1B and Table S1), which contained the 8860 bp WT promoter region of *EFG1* (orf19.610 ), was used as the reference for assessing the effects of the different constructed deletions on RLUC activity in the white and opaque phase (Figure 2). RLUC activity in WT cells in the white phase was more than 10 fold higher than in the opaque phase (Figure 3, A and B). This was consistent with the differential expression of *EFG1* (orf19.610 ) in the white and opaque phases of the parental wild-type strain WO-1 (Sonneborn *et al.* 1999; Srikantha *et al.* 2000). These results indicated that the upstream region of *EFG1* (orf19.610 ) regulated differential *RLUC* expression in the WT reporter construct, just as it regulated *EFG1* (orf19.610 ) expression in the natural strain.

### Effects of deleting most or all of the upstream region

Deletion of the entire upstream region, which includes binding site 1 through 8 (−8856–−2250 bp) in strain ΔP1-8 (Figure 1B), reduced *RLUC* activity in the white phase more than 16 fold (Figure 2A). Deletion of the upstream region that included binding sites 1 through 9 (−8856–−1693 bp) or 1 through the WhTSP (−8856–−1015 bp), in strains ΔP1-9 and ΔP1-WhTSP, reduced RLUC activity in the white phase to negligible levels (Figure 2A). Deletion of the upstream region harboring binding sites 1 through 8 (−8856–−2250 bp), in strain ΔP1-8 (Figure 1B), had no significant effect on the already low level of expression in the opaque phase, and deletion of the region harboring binding sites 1 through 9 (−8856–−1693 bp), in strain ΔP1-9, or site 1 through the WhTSP (−8856–−1015 bp), in strain ΔP1-WhTSP, decreased the already low level of RLUC activity in the opaque phase by approximately 40% (Figure 2C). Computing the ratio of RLUC activity in these major deletion derivatives to that of the WT reporter strain in the white phase, for both the white phase and opaque phase, underscores the conclusion that the contiguous upstream region encompassing binding sites 1 through 8, is essential for approximately 95% of RLUC activity in the white phase, and that the contiguous region that encompasses sites 1 through 9 is essential for close to 100% of RLUC activity (Figure 3A). These results suggest that the contiguous region encompassing sites 1 through 8 is not responsible for suppressing RLUC activity in the opaque phase, and that the region between sites 8 and 9 may actually play an activation role for the low but significant level of activity in the opaque phase.

### Effects of deleting individual binding sites

Of the 10 binding sites identified in the upstream region of *EFG1* (orf19.610 ) (Hernday *et al.* 2013), seven bind one or more of the six network TFs in the white phase and seven bind one or more in the opaque phase (Figure 1A). Four of the sites (1, 6, 8, and 9) bind TFs in both phases (Figure 1A). Individual deletion derivatives were generated for each of the 10 binding sites in strain WO-1 (α/α) (Figure 1B and Table S3). Deletion of any one of the first nine binding sites did not result in a significant decrease in RLUC activity in the white phase. In fact, all of the first nine deletion derivatives but one, ΔP8, exhibited minor increases in RLUC activity (Figure 2B). The increases in RLUC activity of ΔP1, ΔP2, ΔP3, and ΔP4 were significant (p value < 0.05) (Figure 2B, starred bars). The decrease in activity exhibited by ΔP8 was not significant. Individually deleting the region containing WhTSP and P10, in ΔP10, resulted in a 10 fold decrease in RLUC activity in the white phase (Figure 2B and Figure 3B). This was not unexpected given the absence of WhTSP, which is necessary for transcription in the white phase. Moreover, site 10 is within the region encoding the white *EFG1* (orf19.610 ) transcript. It should be noted that site 10 does not bind any of the six network TFs in the white phase (Figure 1A) (Hernday *et al.* 2013).

Individually deleting seven of the 10 binding sites in strain WO-1 resulted in increases in RLUC activity for sites 1, 3, and 4 in the opaque phase (Figure 2D), suggesting that they may play minor roles in suppressing *EFGI* transcription in that phase. The deletions ΔP1, ΔP3, and ΔP4 exhibited 1.3, 1.5, and 2.3 fold WT activity, respectively, which were significant (p value < 0.05) (Figure 2D). Deletion of sites 9 and 10 resulted in significant decreases in RLUC activity (Figure 2D), suggesting that they played *cis*-acting roles as activators of the low, but significant level of *EFG1* (orf19.610 ) expression in the opaque phase.

### Combinatorial deletions

**Figure 4:**
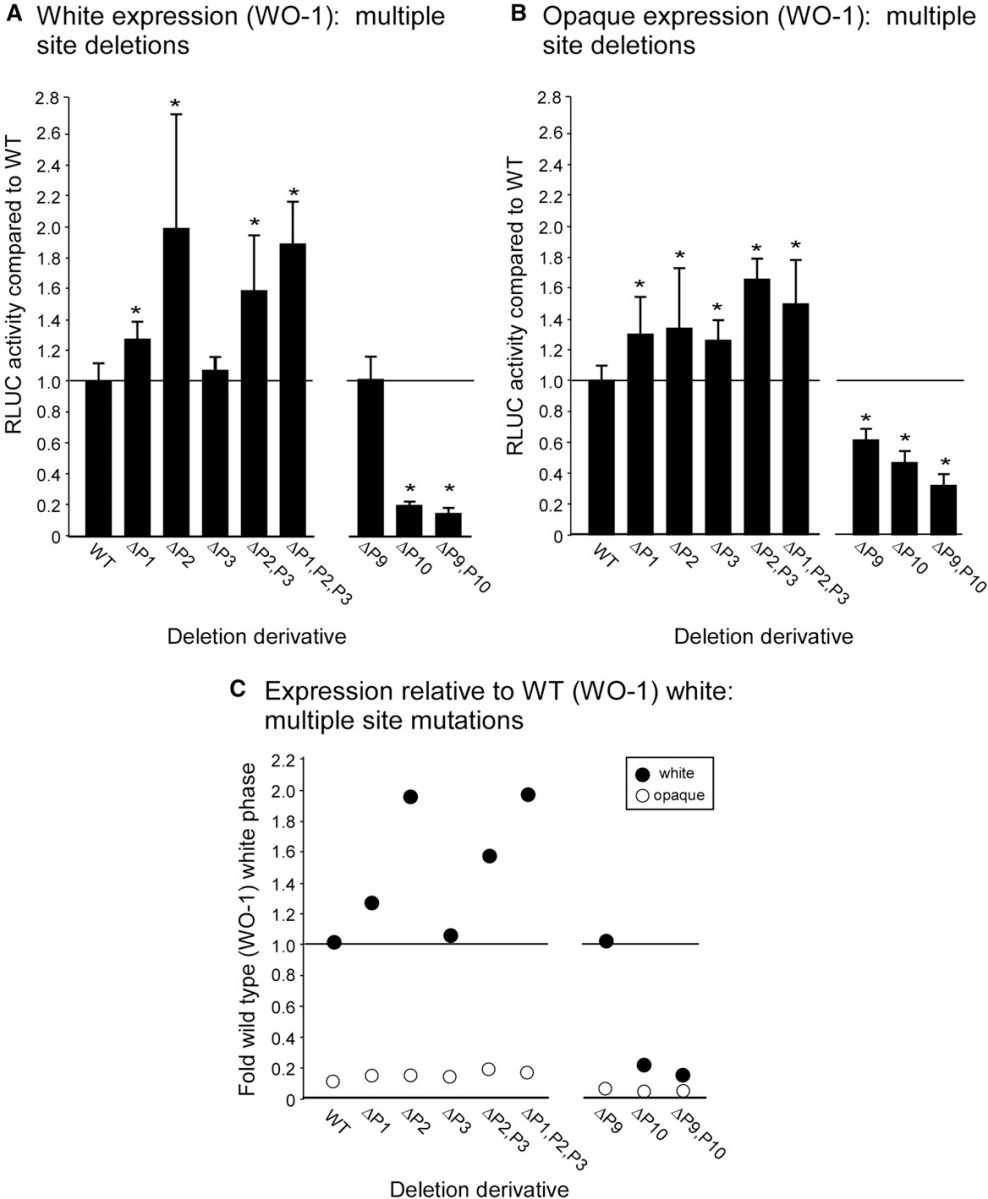
RLUC activity of multiple site deletion derivatives in the α/α strain WO-1. These mutants were not contiguous deletions that included multiple sites, but rather combinations of individual deletions targeting each site in the combination. (A) RLUC activity of targeted deletions ΔP1, ΔP2, ΔP3, ΔP2, ΔP3, and ΔP1,P2,P3, and ΔP9, ΔP10, and ΔP9,P10 in the white phase. (B) RLUC activity in the opaque phase of the targeted deletions listed in panel A. (C) RLUC activity of the derivatives listed in panel A, relative to the activity of the WT derivative in the white phase (p < 0.05; two-tailed Mann–Whitney nonparametric test). RLUC, *Renilla reniformis* luciferase; WT, wild type.

To test whether combinatorial deletions of neighboring binding sites affected RLUC activity, we generated the deletion mutants ΔP2,P3 and ΔP1,P2,P3 (Figure 1B and Table S3). As was the case for individual deletions of sites 1, 2, and 3, neither of the two combinatorial deletion strains exhibited a decrease in RLUC activity in the white phase (Figure 4A), suggesting that sites 1, 2, and 3 do not function additively as enhancers. In fact, ΔP2,P3 and ΔP1,P2,P3, exhibited significant increases in activity of 60% and 80% in the white phase (Figure 4A), respectively, similar to the increases observed for the individual deletions ( Figure 2B and Figure 3B). The increases observed in the former were not larger than the latter, suggesting that these low levels of individual activation were not additive. More importantly, there was no additive decrease in activity in the white phase. In the opaque phase, combinatorial deletion of 2 and 3, or 1, 2, and 3, resulted in significant increases of 1.7 and 1.4 WT activity, respectively, but revealed no additive increases when compared to the RLUC activity of the individual deletions ΔP1, ΔP2, and ΔP3 (Figure 4B). This point is evident when RLUC activities of the mutant strains in the two phases are computed relative to expression in the white phase of the derivative WT, which harbors the full length upstream region of *EFG1* (orf19.610 ) (Figure 4C).

We next generated the combinatorial mutant ΔP9,P10 by individually deleting binding site 9 and the region harboring the WhTSP and site 10, in combination (Figure 1B and Table S3). Strain ΔP9,P10 exhibited the same decrease in RLUC activity in the white phase as ΔP10 (Figure 4A), again noting that the P10 binding site resides within the 5′ end of the region encoding the white phase transcript (Figure 2A). The combinatorial mutant ΔP9,P10 exhibited a decrease in RLUC activity similar to that of the individual deletion derivative ΔP10 in the opaque phase (Figure 4B). It should be noted that the combinatorial mutant ΔP9,P10 harbored the intact OpTSP (Figure 1B).

### Similar results in an a/a strain

**Figure 5:**
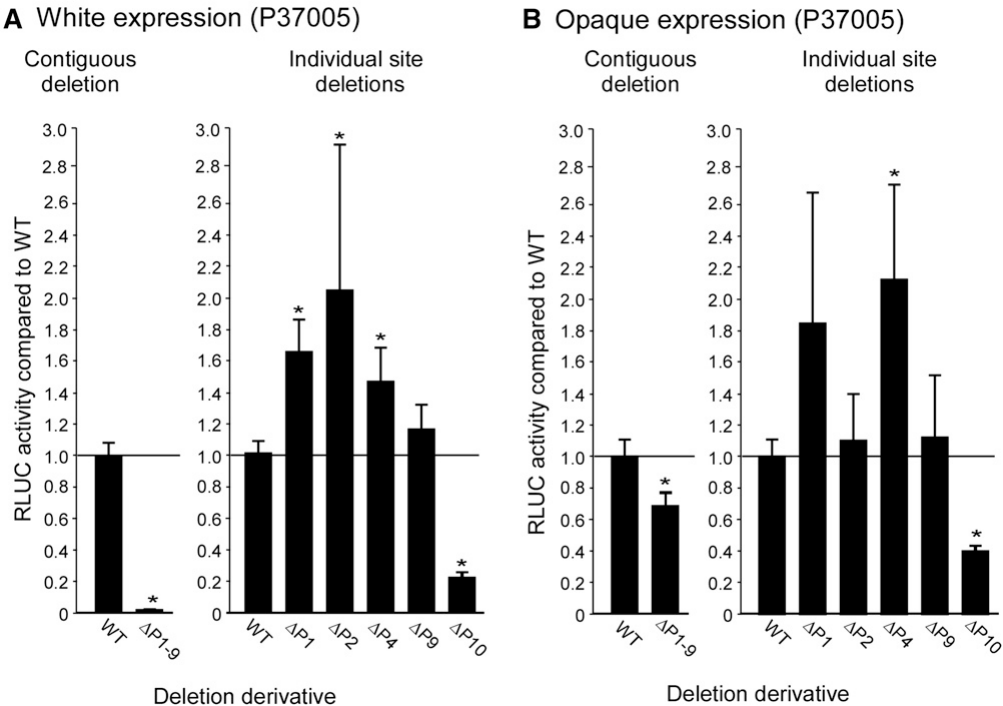
RLUC activity of select deletion derivatives generated in the **a/a** strain P37005, compared to the wild type (WT) derivative containing the full length upstream region of *EFG1*. (A) RLUC activity in the white phase of a contiguous deletion from −8860 bp through binding site 9 (ΔP1-9) and five targeted deletions of transcription factor binding sites. (B) RLUC activity in the opaque phase of the contiguous and targeted deletions (p < 0.05; two-tailed Mann–Whitney nonparametric test). RLUC, *Renilla reniformis* luciferase; WT, wild type.

The preceding deletion analysis was performed with two independently generated strains for each deletion derivative for the parent strain WO-1, which possesses an α/α *MTL* configuration (Lockhart *et al.* 2002; Miller and Johnson 2002). To be sure, however, that our results were not strain or mating type-specific, we selectively generated deletion mutants, including ΔP1-9, ΔP1, ΔP2, ΔP4, ΔP9, and ΔP10, in the natural **a/a** strain P37005 (Lockhart *et al.* 2002). The difference between the white phase and the opaque phase in RLUC activity in the WT derivative of P37005 was 10 fold (data not shown), highly similar to that of the WT derivative of strain WO-1 (α/α) (Figure 3, A and B). Deletion of the entire upstream region harboring binding sites P1 through P9, in strain ΔP1-9, resulted in the nearly complete loss of expression in the white phase (Figure 5A) and a 35% loss in the opaque phase (Figure 5B), results similar to those for the analogous deletion derivatives in strain WO-1 (α/α) (Figure 2, A and C, respectively). Individual deletion of sites 1, 2, 4, and 9 in derivatives ΔP1, ΔP2, ΔP4, and ΔP9 of strain P370005 (**a/a**) did not result in a decrease in RLUC activity in the white phase (Figure 5A). As was the case for analogous deletion derivatives in WO-1 (α/α) (Figure 2B), ΔP1, ΔP2, ΔP4, and ΔP9 resulted in increases in the white phase (Figure 5A). The increases in ΔP1, ΔP2, and ΔP4 were significant. Individual deletion of P1, P2, P4, and P9 resulted in increases in RLUC activity in the opaque phase, between 1.1–2.0 WT activity (Figure 5B). Only the increase in ΔP4 was significant, but the increases as a whole suggested a trend. The trend of increases for sites ΔP1, ΔP2, and ΔP4 correlated with that observed in the parallel deletion derivatives generated in strain WO-1 (**a/a**) (Figure 2D). Deletion of site 10 in ΔP10 caused a major decrease in RLUC activity in the white phase (Figure 5A) and opaque phase (Figure 5B), results again similar to those for the analogous deletion derivative in strain WO-1(α/α) (Figure 2, B and D, respectively). Therefore, the expression data presented for the deletion derivatives of the **a/a** strain P37005 indicate that the more extensive results obtained for strain WO-1 (α/α) were neither strain-specific nor mating type-specific.

## Discussion

Hernday *et al.* (2013) performed a comprehensive study of six TFs involved in the regulation of the white-opaque transition and maintenance of the alternative phenotypes of *C. albicans*. In one component of this study, they employed ChIP-chip analyses to assess binding of the six TFs to sites along the promoters of each TF, and generated models for interactive transcription networks regulating expression of the alternative switch phenotypes. Here, we have explored whether the identified binding sites along the *EFG1* (orf19.610 ) promoter, function as enhancers in the white phase, in which *EFG1* (orf19.610 ) is expressed at high levels, and/or as silencers in the opaque phase, in which *EFG1* (orf19.610 ) is expressed at a 10 fold lower level (Sonneborn *et al.* 1999; Srikantha *et al.* 2000; Zordan *et al.* 2007).

### Assessing binding site function

While ChIP-chip analyses provide a powerful tool for identifying TF binding sites (Harbison *et al.* 2004; Wu *et al.* 2006; Cooke *et al.* 2009; Beyhan *et al.* 2013; Hernday *et al.* 2013; DeVilbiss *et al.* 2014), they do not establish functionality (Li *et al.* 2008; Cooke *et al.* 2009; Ucar *et al.* 2009; Qin *et al.* 2011; Carey *et al.* 2012; Maienschein-Cline *et al.* 2012; Whitfield *et al.* 2012; Nguyen-Duc *et al.* 2013; Teytelman *et al.* 2013; Wei *et al.* 2013; Wu and Ji 2013; Cusanovich *et al.* 2014; DeVilbiss *et al.* 2014; Sanalkumar *et al.* 2014; Slattery *et al.* 2014; Bansal *et al.* 2015). A variety of studies have shown this to be the case. Ucar *et al.* (2009), integrated ChIP-chip binding data with motif binding sites, nucleosome occupancy, and mRNA expression profiles in *Saccharomyces cerevisiae*, and estimated that approximately 50% of protein–DNA binding interactions may be nonfunctional. Whitfield *et al.* (2012) analyzed 455 binding sites in four immortalized cell lines using a transient transfection strategy with a luciferase reporter assay. In each cell line, only 36–49% of binding sites of the promoter tested were deemed functional. Nguyen-Duc *et al.* (2013) employed ChIP-chip to map binding of the leucine-responsive TF, Ss-LrpB, in *Sulfolobus solfataricus*, an archaebacterium. They identified 36 loci in addition to four already known that bind Ss-LrpB. However, by comparing the transcription profiles of the wild type and the *Ss-LrpB* deletion mutant, they found that out of 12 tested genes, only one, *CRISPR B*, was regulated by Ss-LrpB. In that study, electrophoretic mobility shift assays failed to correlate with the ChIP-chip data, suggesting possible artifacts in the latter technology. Wei *et al.* (2013) estimated that only 25% of the binding sites identified by the TF MYC in ChIP-seq analyses were potentially functional targets, based on transcription profiles in MYC perturbation experiments. It should be noted, however, that transcriptional analysis of target genes in TF mutants or cells in which RNAi is used to downregulate the TF do not test the possibility of TF redundancy whenever a potential target is not affected (Spivakov 2014).

Another approach to assess the functionality of TF binding interactions is to delete the binding sites. Meyer *et al.* (2010) found that the vitamin D and retinoid X receptors, which form a heterodimer, bind to six sites located upstream and downstream of the gene *CYP24A1*, in the presence of vitamin D. A deletion analysis of the sites revealed that both the one site upstream and five downstream were involved in upregulating *CYP24A1*, and, most importantly, that their individual functions were additive. Bresnick and colleagues (DeVilbiss *et al.* 2014; Sanalkumar *et al.* 2014) employed ChIP-seq to identify four GATA-1/2 binding sites close to the *Gata2* gene, three upstream and one in an intron. Targeted deletions in this case revealed that the three in the upstream region appeared to play no role in the regulation of *Gata2* during hematopoiesis, but the site in the intron played a major role. One of the most interesting analyses assessing function of binding sites by targeted deletions involves the silent *MTL* of *S. cerevisiae*, *HMR* and *HML*. Both are flanked by an essential silencer (E-silencer) and an important silencer (I-silencer). Deletion of the Abf1 or Orc1 binding site in HMR-E individually had no effect on *HMR* repression, and deletion of the Rap1 binding site had a 10% effect on repression (Brand *et al.* 1987). However, combinatorial deletion of the Abf1 and Rap1 binding sites, or the Rap1 and Orc1 binding sites, completely derepressed *HMR* expression. Such combinatorial effects indicate redundancy and can reflect cooperativity, most likely in chromatin looping to bring regulatory elements to transcription start points (Koch *et al.* 1989; Kadauke and Blobel 2009; Cao *et al.* 2014). An example of additivity and redundancy can also be found in *HML* regulation (Mahoney *et al.* 1991). Deleting the Sum1 binding site in HML-E resulted in 15% derepression, deleting the Rap1 binding site resulted in 28% derepression, and deleting the Orc1 binding site resulted in 61% derepression. Combinatorial deletions of the Rap1 and Orc1 binding sites or the Orc1 and Sum1 binding sites resulted in 100% derepression, suggesting both additivity and redundancy. The results for HMR-E (Brand *et al.* 1987) demonstrate a case of redundancy in which individually deleting binding sites may not be sufficient to identify function. In addition to these caveats in assessing binding site function, ChIP-chip analyses can be problematic. Teytelman *et al.* (2009) found that chromatin structure can affect the immunoprecipitation step, given that silenced chromatin can be refractory to shearing, thus biasing binding to sites in genes differentially expressed in alternative phenotypes. Teytelman *et al.* (2013) have also presented evidence that regions highly expressed can bind TFs nonspecifically, which they have referred to as “hyper-ChIPable.”

### Deletion analysis of the EFG1 promoter

It is, therefore, critical to demonstrate that TF binding sites identified by ChIP-chip or ChIP-seq are functional, but as Carey *et al.* (2012) have carefully considered, there is no single experimental strategy that definitively demonstrates functionality. They describe 12 strategies to demonstrate that a protein–DNA interaction may be functional. Here, we have employed the strategy of targeted deletions to begin to assess the functionality of the 10 sites that bind network TFs along the promoter of *EFG1* (orf19.610 ), a major regulator of the white phase phenotype. It should be emphasized that this study represents the first of several steps, after ChIP-chip analysis (Hernday *et al.* 2013), in understanding how *EFG1* (orf19.610 ) is regulated.

Because *EFG1* (orf19.610 ) transcription is 10 fold higher in the white phase and is essential for full expression of the white phase, we expected to find that one or more of the seven sites upstream of *EFG1* (orf19.610 ) that bind network TFs in the white phase function as *cis*-acting enhancers, and/or that one or more of the seven sites upstream of *EFG1* (orf19.610 ) that bind network TFs in the opaque phase function as *cis*-acting silencers in the opaque phase. If one or more of the seven identified sites (1, 2, 3, 6, 7, 8, 9) that bind network TFs in the white phase acted independently as a *cis*-acting enhancer, and their functions were additive, then a targeted deletion of that site would result in a decrease in RLUC activity. None of the individual targeted deletions of the white phase binding sites, ΔP1, ΔP2, ΔP3, ΔP6, ΔP7, ΔP8, and ΔP9 generated in strain WO-1(α/α), and none of the targeted deletions, ΔP1, ΔP2, ΔP4, and ΔP9 generated in strain P37005 (**a**/**a**), reduced RLUC activity significantly in the white phase. In fact, all but one of the targeted single deletions exhibited small increases in RLUC activity, and the decrease observed in ΔP8 was not significant. These increases could be due to alterations in chromatin architecture resulting from the deletions. The absence of decreases, however, do not support models of additivity of the bound sites in the enhancement of *EFG1* (orf19.610 ) expression in the white phase. Combinatorial deletion of binding sites 2 and 3, and 1, 2, and 3, again resulted in similar increases, not decreases, in RLUC activity in the white phase, supporting the preceding interpretation. The combinatorial deletions exhibited roughly the same small increases as the individual deletions. However, a contiguous deletion from −8860 bp through binding site 8 (ΔP1-8), from −8860 bp through site 9 (ΔP1-9), and from −8860 bp through the white TSP (ΔP1-WhTSP), reduced RLUC activity to the low level observed in the opaque phase, indicating that the elevated level of *EFG1* (orf19.610 ) expression in the white phase is dependent upon a relatively intact promoter and, therefore, sites in that promoter. The results of ΔP7 and ΔP8, which bind only TF Ahr1, are consistent with those of Hernday *et al.* (2013), who found that deletion of *AHR1* (orf19.7381 ) does not affect the level of expression of *EFG1* (orf19.610 ) in the white phase.

If one or more of the seven sites (sites 1, 4, 5, 6, 8, 9, and 10) that bind network TFs in the opaque phase acted independently and additively as *cis*-acting silencers, then a targeted deletion of that site would result in an increase in RLUC activity. Deletion of sites 1, 2, 3, and 4 did result in increases in RLUC activity in the opaque phase. The increases ranged between 5–10% of white phase WT expression. The combinatorial deletions of sites 1, 2, and 3, however, did not show additivity, resulting in an increase of only 5% of WT white phase expression. These results therefore provide no support for additivity. Deletion of binding sites 9 and 10 resulted in RLUC activities in the opaque phase that were actually 0.5 and 0.2 that of the WT derivative in the opaque phase, respectively, suggesting that rather than playing roles in silencing, these sites played roles in enhancing the low but significant level of RLUC expression in the opaque phase. The individual deletion that resulted in the largest increase in RLUC activity in the opaque phase, approximately 10% of the WT white phase level, was that of ΔP4. Consistent with this observation, Hernday *et al.* (2013) found that deleting *CZF1* (orf19.3127 ), which binds to site 4 in the opaque phase, results in an increase in *EFG1* (orf19.610 ) expression in the opaque phase. Interestingly, mutating *WOR3* (orf19.467 ), which also binds site 4, does not result in an increase in *EFG1* (orf19.610 ) expression (Hernday *et al.* 2013; Lohse *et al.* 2013), and neither deletion of site 6 nor deletion of site 9, both of which bind Czf1 in the opaque phase, resulted in an increase in RLUC activity in the opaque phase. When the contiguous region from −8860 bp through binding site 8 was deleted, the low level of RLUC expression in the opaque phase did not change (*i.e.*, increase). This suggests that the contiguous upstream region may not play a general silencing role in the low but significant level of *EFG1* (orf19.610 ) expression in the opaque phase.

### Previous mutational analyses of network TFs

A minimal expectation for a functional role of a network TF in the regulation of *EFG1* (orf19.610 ) expression would be that deletion of a TF regulating *EFG1* (orf19.610 ) would indeed have an effect on *EFG1* (orf19.610 ) expression (Cooke *et al.* 2009; Qin *et al.* 2011; Maienschein-Cline *et al.* 2012; Wei *et al.* 2013; Wu and Ji 2013). However, this is by no means a definitive test since it does not assess TF redundancy (Spivakov 2014). An analysis of the effects of mutating network TFs on *EFG1* (orf19.610 ) expression has been performed for *WOR3* (orf19.467 ), *AHR1* (orf19.7381 ), and *CZF1* (orf19.3127 ), but not for *WOR2* (orf19.5992 ) or *WOR1* (orf19.4884 ). Deletion of *WOR3* (orf19.467 ) did not affect differential expression of *EFG1* (orf19.610 ) in the alternative phases (Hernday *et al.* 2013; Lohse *et al.* 2013). Wor3 binds at sites 4, 6, and 10 along the *EFG1* (orf19.610 ) promoter in the opaque phase (Figure 2) (Hernday *et al.* 2013). Since these sites bind several network TFs, the absence of an effect in a TF deletion mutant does not definitively exclude functionality if TF redundancy exists (Spivakov 2014). Deletion of *AHR1* (orf19.7381 ) also does not affect the differential expression of *EFG1* (orf19.610 ) in the alternative phases (Hernday *et al.* 2013). Ahr1 binds to sites 7 and 8 in the white phase and 5 and 8 in the opaque phase (Hernday *et al.* 2013). For the two sites in the white phase and the two sites in the opaque phase, Ahr1 is the only network TF that binds, and hence Ahr1 binding alone defines the sites. Since other network TFs do not bind these sites, the lack of an effect by *AHR1* (orf19.7381 ) mutation cannot at this time be explained by TF redundancy, although it is possible that TFs other than the identified network TFs, bind to these sites and function redundantly. The deletion of *CZF1* (orf19.3127 ), however, resulted in an increase in the expression of *EFG1* (orf19.610 ) in the opaque phase (Hernday *et al.* 2013). Czf1 binds site 9 in the white phase and sites 4, 6, and 9 in the opaque phase (Hernday *et al.* 2013). The deletion of site 4 results in an increase in expression in the opaque phase. All of the sites Czf1 binds to include binding by other network TFs. Hence, TF redundancy cannot be ruled out at site 4. Therefore, the increase in *EFG1* (orf19.610 ) expression in the opaque phase of the *CZF1* (orf19.3127 ) mutant (Hernday *et al.* 2013) may be more consistent with an indirect role for *CZF1* (orf19.3127 ) in the regulation of *EFG1* (orf19.610 ). Finally, our results, using targeted deletion of individual sites, suggest that sites 1, 2, 3, and 4 play a role in partially suppressing *EFG1* (orf19.610 ) expression in the opaque phase. However, in the opaque phase only site 4 binds network TFs, Wor3 and Czf1, that were analyzed by gene mutation, and only the latter mutant showed an increase in *EFG1* (orf19.610 ) expression in the opaque phase. It can reasonably be concluded that individual TF mutants add little to the interpretation of network TF binding site functionality at the *EFG1* (orf19.610 ) promoter.

### Concluding remarks

Deletion of network TF binding sites along the *EFG1* (orf19.610 ) promoter failed to provide strong evidence for individual *cis*-acting enhancement roles in the white phase or *cis*-acting silencing roles in the opaque phase, except possibly for silencer activity of sites 1, 2, 3, and 4 in the opaque phase. Combinatorial deletion of sites 1, 2, and 3, failed to reveal cooperative or additive enhancer roles in the white phase or additive silencer roles in the opaque phase. The results of individual, combinatorial, or extensive deletions revealed no indication of suppression of *EFG1* (orf19.610 ) expression by the promoter in the opaque phase. These results are consistent with, but not definitive for, the conclusion that the majority of binding sites may be nonfunctional. There are several alternative explanations for these results that do not exclude functionality. First, it must be noted that in the present study, regulation was limited to log phase growth in the alternative phenotypes under a single set of conditions. Regulation during the actual phenotypic transition was not assessed. This was also true for the study by Hernday *et al.* (2013) which identified the binding sites. Hence, the functionality of the sites in the enhancement of *EFG1* (orf19.610 ) expression in the opaque to white transition or silencing in the white to opaque transition were not assessed. Rather, steady state regulation in the established alternative phenotypes was assessed. Moreover, *EFG1* (orf19.610 ) is differentially regulated during formation of hyphae (Stoldt *et al.* 1997), and both white and opaque cells form hyphae (Anderson *et al.* 1989). Therefore, experiments are now in progress to test whether the identified binding sites function during the actual phenotypic transitions accompanying white-opaque switching or during hyphal formation. Second, the binding sites may function under different nutritional or environmental conditions to those used here to maintain the white and opaque phases (Harbison *et al.* 2004; Ucar *et al.* 2009; Spivakov 2014). Third, although the results of our study do not reveal cooperativity or additivity among network TF binding sites in the process of activation in the white phase, they do not definitively rule them out because the combinatorial deletions performed here were not extensive enough. Our results also do not rule out binding site redundancy for the same reason. However, there is no indication that the binding sites play silencing roles in the low level of expression in the opaque phase. In this case, the expression levels in the opaque phase, for ΔP1-8, ΔP1-9, and ΔP1-TSP, were even lower than the already low level of expression in this phase in the WT construct. None of our data exclude the possibility that other functional sites, bound by nonnetwork TFs, are responsible for activation of *EFG1* (orf19.610 ) in the white phase or the more remote possibility of repression in the opaque phase. An even more detailed functional analysis, including a more comprehensive set of combinatorial deletions and strategies (Carey *et al.* 2012), will be required to fully elucidate which sites or regions of the promoter of *EFG1* (orf19.610 ) and which TFs regulate expression.

## 

## Supplementary Material

Supplemental Material

## References

[bib1] Anderson J. Cundiff L. Schnars B. Gao M. X. Mackenzie I., 1989 Hypha formation in the white-opaque transition of *Candida albicans*. Infect. Immun. 57: 458–467. 2643570 10.1128/iai.57.2.458-467.1989 PMC313119

[bib2] Anderson J. M. Soll D. R., 1987 Unique phenotype of opaque cells in the white-opaque transition of Candida albicans. J. Bacteriol. 169: 5579–5588. 3316187 10.1128/jb.169.12.5579-5588.1987 PMC213989

[bib3] Bansal M. Mendiratta G. Anand S. Kushwaha R. Kim R. H., 2015 Direct ChIP-Seq significance analysis improves target prediction. BMC Genomics 16: S4. 26040656 10.1186/1471-2164-16-S5-S4 PMC4460594

[bib4] Basma R. Barada G. Ojaimi N. Khalaf R. A., 2009 Susceptibility of *Candida albicans* to common and novel antifungal drugs, and relationship between the mating type locus and resistance, in Lebanese hospital isolates. Mycoses 52: 141–148. 18627469 10.1111/j.1439-0507.2008.01559.x

[bib5] Basso L. R. Jr Bartiss A. Mao Y. Gast C. E. Coelho P. S., 2010 Transformation of *Candida albicans* with a synthetic hygromycin B resistance gene. Yeast 27: 1039–1048. 20737428 10.1002/yea.1813 PMC4243612

[bib6] Bedell G. W. Soll D. R., 1979 Effects of low concentrations of zinc on the growth and dimorphism of *Candida albicans*: evidence for zinc-resistant and-sensitive pathways for mycelium formation. Infect. Immun. 26: 348–354. 387610 10.1128/iai.26.1.348-354.1979 PMC414618

[bib7] Berkes, C. A., and S. J. Tapscott, 2005 MyoD and the transcriptional control of myogenesis. Semin. Cell Dev. Biol. 16: 585–595. 10.1016/j.semcdb.2005.07.006 16099183

[bib8] Beyhan S. Gutierrez M. Voorhies M. Sil A., 2013 A temperature-responsive network links cell shape and virulence traits in a primary fungal pathogen. PLoS Biol. 11: e1001614. 23935449 10.1371/journal.pbio.1001614 PMC3720256

[bib9] Brand A. H. Micklem G. Nasmyth K., 1987 A yeast silencer contains sequences that can promote autonomous plasmid replication and transcriptional activation. Cell 51: 709–719. 3315230 10.1016/0092-8674(87)90094-8

[bib10] Cao S. Kumimoto R. W. Gnesutta N. Calogero A. M. Mantovani R., 2014 A distal *CCAAT*/nuclear factor Y complex promotes chromatin looping at the *FLOWERING LOCUS T* promoter and regulates the timing of flowering in *Arabidopsis*. Plant Cell 26: 1009–1017. 24610724 10.1105/tpc.113.120352 PMC4001365

[bib11] Carey M. F. Peterson C. L. Smale S. T., 2012 Confirming the functional importance of a protein-DNA interaction. Cold Spring Harb. Protoc. 2012: 733–757. 22753608 10.1101/pdb.top070060

[bib12] Cho B.-K. Federowicz S. Park Y.-S. Zengler K. Palsson B. Ø., 2012 Deciphering the transcriptional regulatory logic of amino acid metabolism. Nat. Chem. Biol. 8: 65–71. 22082910 10.1038/nchembio.710 PMC3777760

[bib13] Cooke E. J. Savage R. S. Wild D. L., 2009 Computational approaches to the integration of gene expression, ChIP-chip and sequence data in the inference of gene regulatory networks. Semin. Cell Dev. Biol. 20: 863–868. 19682595 10.1016/j.semcdb.2009.08.004

[bib14] Cusanovich D. A. Pavlovic B. Pritchard J. K. Gilad Y., 2014 The functional consequences of variation in transcription factor binding. PLoS Genet. 10: e1004226. 24603674 10.1371/journal.pgen.1004226 PMC3945204

[bib15] Daniels K. J. Srikantha T. Lockhart S. R. Pujol C. Soll D. R., 2006 Opaque cells signal white cells to form biofilms in *Candida albicans*. EMBO J. 25: 2240–2252. 16628217 10.1038/sj.emboj.7601099 PMC1462973

[bib16] De Backer M. D. Maes D. Vandoninck S. Logghe M. Contreras R., 1999 Transformation of *Candida albicans* by electroporation. Yeast 15: 1609–1618. 10572258 10.1002/(sici)1097-0061(199911)15:15<1609::aid-yea485>3.3.co;2-p

[bib17] DeVilbiss A. W. Sanalkumar R. Johnson K. D. Keles S. Bresnick E. H., 2014 Hematopoietic transcriptional mechanisms: from locus-specific to genome-wide vantage points. Exp. Hematol. 42: 618–629. 24816274 10.1016/j.exphem.2014.05.004 PMC4125519

[bib18] Federowicz S. Kim D. Ebrahim A. Lerman J. Nagarajan H., 2014 Determining the control circuitry of redox metabolism at the genome-scale. PLoS Genet. 10: e1004264. 24699140 10.1371/journal.pgen.1004264 PMC3974632

[bib19] Harbison C. T. Gordon D. B. Lee T. I. Rinaldi N. J. Macisaac K. D., 2004 Transcriptional regulatory code of a eukaryotic genome. Nature 431: 99–104. 15343339 10.1038/nature02800 PMC3006441

[bib20] Hernday A. D. Lohse M. B. Fordyce P. M. Nobile C. J. DeRisi J. L., 2013 Structure of the transcriptional network controlling white‐opaque switching in *Candida albicans*. Mol. Microbiol. 90: 22–35. 23855748 10.1111/mmi.12329 PMC3888361

[bib21] Hobson R., 2003 The global epidemiology of invasive Candida infections—is the tide turning? J. Hosp. Infect. 55: 159–168. 14572481 10.1016/j.jhin.2003.08.012

[bib22] Hu P. Geles K. G. Paik J.-H. DePinho R. A. Tjian R., 2008 Codependent activators direct myoblast-specific MyoD transcription. Dev. Cell 15: 534–546. 18854138 10.1016/j.devcel.2008.08.018 PMC2614327

[bib23] Huang G. Wang H. Chou S. Nie X. Chen J., 2006 Bistable expression of *WOR1*, a master regulator of white–opaque switching in *Candida albicans*. Proc. Natl. Acad. Sci. USA 103: 12813–12818. 16905649 10.1073/pnas.0605270103 PMC1540355

[bib24] Huffnagle G. B. Noverr M. C., 2013 The emerging world of the fungal microbiome. Trends Microbiol. 21: 334–341. 23685069 10.1016/j.tim.2013.04.002 PMC3708484

[bib25] Hull C. M. Johnson A. D., 1999 Identification of a mating type-like locus in the asexual pathogenic yeast *Candida albicans*. Science 285: 1271–1275. 10455055 10.1126/science.285.5431.1271

[bib26] Hull C. M. Raisner R. M. Johnson A. D., 2000 Evidence for mating of the” asexual” yeast *Candida albicans* in a mammalian host. Science 289: 307–310. 10894780 10.1126/science.289.5477.307

[bib27] Kadauke S. Blobel G. A., 2009 Chromatin loops in gene regulation. Biochim. Biophys. Acta 1789: 17–25. 18675948 10.1016/j.bbagrm.2008.07.002 PMC2638769

[bib28] Koch W. Benoist C. Mathis D., 1989 Anatomy of a new B-cell-specific enhancer. Mol. Cell. Biol. 9: 303–311. 2467189 10.1128/mcb.9.1.303-311.1989 PMC362173

[bib29] Kwon E.-J. G. Laderoute A. Chatfield-Reed K. Vachon L. Karagiannis J., 2012 Deciphering the transcriptional-regulatory network of flocculation in *Schizosaccharomyces pombe*. PLoS Genet. 8: e1003104. 23236291 10.1371/journal.pgen.1003104 PMC3516552

[bib30] Lachke S. A. Srikantha T. Soll D. R., 2003 The regulation of *EFG1* in white–opaque switching in *Candida albicans* involves overlapping promoters. Mol. Microbiol. 48: 523–536. 12675809 10.1046/j.1365-2958.2003.t01-1-03448.x

[bib31] Lee K. Buckley H. R. Campbell C. C., 1975 An amino acid liquid synthetic medium for the development of mycellal and yeast forms of *Candida albicans*. Med. Mycol. 13: 148–153. 10.1080/00362177585190271 808868

[bib32] Legrand M. Lephart P. Forche A. Mueller F. M. C. Walsh T., 2004 Homozygosity at the MTL locus in clinical strains of *Candida albicans*: karyotypic rearrangements and tetraploid formation†. Mol. Microbiol. 52: 1451–1462. 15165246 10.1111/j.1365-2958.2004.04068.x

[bib33] Li X. Y. MacArthur S. Bourgon R. Nix D. Pollard D. A., 2008 Transcription factors bind thousands of active and inactive regions in the *Drosophila* blastoderm. PLoS Biol. 6: e27. 18271625 10.1371/journal.pbio.0060027 PMC2235902

[bib34] Lin C.-C. Yang C.-H. Lin Y.-J. Chiu Y.-W. Chen C.-Y., 2015 Establishment of a melanogenesis regulation assay system using a fluorescent protein reporter combined with the promoters for the melanogenesis-related genes in human melanoma cells. Enzyme Microb. Technol. 68: 1–9. 25435499 10.1016/j.enzmictec.2014.09.008

[bib35] Lockhart S. R. Pujol C. Daniels K. J. Miller M. G. Johnson A. D., 2002 In *Candida albicans*, white-opaque switchers are homozygous for mating type. Genetics 162: 737–745. 12399384 10.1093/genetics/162.2.737 PMC1462282

[bib36] Lockhart S. R. Daniels K. J. Zhao R. Wessels D. Soll D. R., 2003 Cell biology of mating in *Candida albicans*. Eukaryot. Cell 2: 49–61. 12582122 10.1128/EC.2.1.49-61.2003 PMC141171

[bib37] Lohse M. B. Hernday A. D. Fordyce P. M. Noiman L. Sorrells T. R., 2013 Identification and characterization of a previously undescribed family of sequence-specific DNA-binding domains. Proc. Natl. Acad. Sci. USA 110: 7660–7665. 23610392 10.1073/pnas.1221734110 PMC3651432

[bib38] Magee B. Magee P., 2000 Induction of mating in *Candida albicans* by construction of *MTL***a** and *MTL*α strains. Science 289: 310–313. 10894781 10.1126/science.289.5477.310

[bib39] Mahoney D. J. Marquardt R. Shei G. J. Rose A. B. Broach J. R., 1991 Mutations in the *HML* E silencer of *Saccharomyces cerevisiae* yield metastable inheritance of transcriptional repression. Genes Dev. 5: 605–615. 2010086 10.1101/gad.5.4.605

[bib40] Maienschein-Cline M. Zhou J. White K. P. Sciammas R. Dinner A. R., 2012 Discovering transcription factor regulatory targets using gene expression and binding data. Bioinformatics 28: 206–213. 22084256 10.1093/bioinformatics/btr628 PMC3259433

[bib41] McManus B. A. Coleman D. C., 2014 Molecular epidemiology, phylogeny and evolution of *Candida albicans*. Infect. Genet. Evol. 21: 166–178. 24269341 10.1016/j.meegid.2013.11.008

[bib42] Meyer M. B. Goetsch P. D. Pike J. W., 2010 A downstream intergenic cluster of regulatory enhancers contributes to the induction of *CYP24A1* expression by 1α, 25-dihydroxyvitamin D3. J. Biol. Chem. 285: 15599–15610. 20236932 10.1074/jbc.M110.119958 PMC2865326

[bib43] Miller M. G. Johnson A. D., 2002 White-opaque switching in *Candida albicans* is controlled by mating-type locus homeodomain proteins and allows efficient mating. Cell 110: 293–302. 12176317 10.1016/s0092-8674(02)00837-1

[bib44] Mönke G. Seifert M. Keilwagen J. Mohr M. Grosse I., 2012 Toward the identification and regulation of the *Arabidopsis thaliana* ABI3 regulon. Nucleic Acids Res. 40: 8240–8254. 22730287 10.1093/nar/gks594 PMC3458547

[bib45] Nguyen-Duc T. van Oeffelen L. Song N. Hassanzadeh-Ghassabeh G. Muyldermans S., 2013 The genome-wide binding profile of the *Sulfolobus solfataricus* transcription factor Ss-LrpB shows binding events beyond direct transcription regulation. BMC Genomics 14: 828. 24274039 10.1186/1471-2164-14-828 PMC4046817

[bib46] Odds F. C., 1988 Candida and candidosis: a review and bibliography, Bailliere Tindall, Sidcup.

[bib47] Odds F. C., 1998 Should resistance to azole antifungals in vitro be interpreted as predicting clinical non-response? Drug Resist. Updat. 1: 11–15. 17092791 10.1016/s1368-7646(98)80209-4

[bib48] Odds F. C. Bougnoux M.-E. Shaw D. J. Bain J. M. Davidson A. D., 2007 Molecular phylogenetics of *Candida albicans*. Eukaryot. Cell 6: 1041–1052. 17416899 10.1128/EC.00041-07 PMC1951527

[bib49] Park Y.-N. Morschhäuser J., 2005 Tetracycline-inducible gene expression and gene deletion in *Candida albicans*. Eukaryot. Cell 4: 1328–1342. 16087738 10.1128/EC.4.8.1328-1342.2005 PMC1214539

[bib50] Park Y.-N. Daniels K. J. Pujol C. Srikantha T. Soll D. R., 2013 *Candida albicans* forms a specialized “sexual” as well as “pathogenic” biofilm. Eukaryot. Cell 12: 1120–1131. 23771904 10.1128/EC.00112-13 PMC3754541

[bib51] Pfaller M. Diekema D., 2007 Epidemiology of invasive candidiasis: a persistent public health problem. Clin. Microbiol. Rev. 20: 133–163. 17223626 10.1128/CMR.00029-06 PMC1797637

[bib52] Qin J. Li M. J. Wang P. Zhang M. Q. Wang J., 2011 ChIP-Array: combinatory analysis of ChIP-seq/chip and microarray gene expression data to discover direct/indirect targets of a transcription factor. Nucleic Acids Res. 39: W430–W436. 21586587 10.1093/nar/gkr332 PMC3125757

[bib53] Reuß O. Vik Å. Kolter R. Morschhäuser J., 2004 The *SAT1* flipper, an optimized tool for gene disruption in *Candida albicans*. Gene 341: 119–127. 15474295 10.1016/j.gene.2004.06.021

[bib54] Rustad T. R. Stevens D. A. Pfaller M. A. White T. C., 2002 Homozygosity at the *Candida albicans MTL* locus associated with azole resistance. Microbiol. 148: 1061–1072. 10.1099/00221287-148-4-1061 11932451

[bib55] Sanalkumar R. Johnson K. D. Gao X. Boyer M. E. Chang Y.-I., 2014 Mechanism governing a stem cell-generating cis-regulatory element. Proc. Natl. Acad. Sci. USA 111: E1091–E1100. 24616499 10.1073/pnas.1400065111 PMC3970491

[bib56] Slattery M. Zhou T. Yang L. Dantas Machado A. C. Gordan R., 2014 Absence of a simple code: how transcription factors read the genome. Trends Biochem. Sci. 39: 381–399. 25129887 10.1016/j.tibs.2014.07.002 PMC4149858

[bib57] Slutsky B. Staebell M. Anderson J. Risen L. Pfaller M., 1987 “White-opaque transition”: a second high-frequency switching system in *Candida albicans*. J. Bacteriol. 169: 189–197. 3539914 10.1128/jb.169.1.189-197.1987 PMC211752

[bib58] Soll D. R., 2014 The role of phenotypic switching in the basic biology and pathogenesis of *Candida albicans*. J. Oral Microbiol. 6: 22993. 10.3402/jom.v6.22993 PMC3895265 24455104

[bib59] Sonneborn A. Tebarth B. Ernst J. F., 1999 Control of white-opaque phenotypic switching in *Candida albicans* by the Efg1p morphogenetic regulator. Infect. Immun. 67: 4655–4660. 10456912 10.1128/iai.67.9.4655-4660.1999 PMC96790

[bib60] Spivakov M., 2014 Spurious transcription factor binding: non-functional or genetically redundant? BioEssays 36: 798–806. 24888900 10.1002/bies.201400036 PMC4230394

[bib61] Srikantha T. Klapach A. Lorenz W. W. Tsai L. K. Laughlin L. A., 1996 The sea pansy *Renilla reniformis* luciferase serves as a sensitive bioluminescent reporter for differential gene expression in *Candida albicans*. J. Bacteriol. 178: 121–129. 8550405 10.1128/jb.178.1.121-129.1996 PMC177628

[bib62] Srikantha T. Tsai L. K. Daniels K. Soll D. R., 2000 *EFG1* null mutants of *Candida albicans* switch but cannot express the complete phenotype of white-phase budding cells. J. Bacteriol. 182: 1580–1591. 10692363 10.1128/jb.182.6.1580-1591.2000 PMC94455

[bib63] Srikantha T. Borneman A. R. Daniels K. J. Pujol C. Wu W., 2006 *TOS9* regulates white-opaque switching in *Candida albicans*. Eukaryot. Cell 5: 1674–1687. 16950924 10.1128/EC.00252-06 PMC1595353

[bib64] Srikantha T. Daniels K. J. Pujol C. Sahni N. Yi S., 2012 Nonsex genes in the mating type locus of *Candida albicans* play roles in **a**/α biofilm formation, including impermeability and fluconazole resistance. PLoS Pathog. 8: e1002476. 22253594 10.1371/journal.ppat.1002476 PMC3257300

[bib65] Srikantha T. Daniels K. J. Pujol C. Kim E. Soll D. R., 2013 Identification of genes upregulated by the transcription factor Bcr1 that are involved in impermeability, impenetrability, and drug resistance of *Candida albicans* **a**/α biofilms. Eukaryot. Cell 12: 875–888. 23563485 10.1128/EC.00071-13 PMC3675989

[bib66] Stoldt V. R. Sonneborn A. Leuker C. E. Ernst J. F., 1997 Efg1p, an essential regulator of morphogenesis of the human pathogen *Candida albicans*, is a member of a conserved class of bHLH proteins regulating morphogenetic processes in fungi. EMBO J. 16: 1982–1991. 9155024 10.1093/emboj/16.8.1982 PMC1169801

[bib67] Sun Y. Fan X.-Y. Cao D.-M. Tang W. He K., 2010 Integration of brassinosteroid signal transduction with the transcription network for plant growth regulation in Arabidopsis. Dev. Cell 19: 765–777. 21074725 10.1016/j.devcel.2010.10.010 PMC3018842

[bib68] Suzuki A. Fujii H. Hoshida H. Akada R., 2015 Gene expression analysis using strains constructed by NHEJ-mediated one-step promoter cloning in the yeast *Kluyveromyces marxianus*. FEMS Yeast Res. 15: fov059. 26136515 10.1093/femsyr/fov059

[bib69] Taka S. Gazouli M. Sotirakoglou K. Liandris E. Andreadou M., 2015 Functional analysis of 3′UTR polymorphisms in the caprine *SLC11A1* gene and its association with the *Mycobacterium avium* subsp. *paratuberculosis* infection. Vet. Immunol. Immunopathol. 167: 75–79. 26117376 10.1016/j.vetimm.2015.06.004

[bib70] Teytelman L. Ozaydin B. Zill O. Lefrancois P. Snyder M., 2009 Impact of chromatin structures on DNA processing for genomic analyses. PLoS One 4: e6700. 19693276 10.1371/journal.pone.0006700 PMC2725323

[bib71] Teytelman L. Thurtle D. M. Rine J. van Oudenaarden A., 2013 Highly expressed loci are vulnerable to misleading ChIP localization of multiple unrelated proteins. Proc. Natl. Acad. Sci. USA 110: 18602–18607. 24173036 10.1073/pnas.1316064110 PMC3831989

[bib72] Tong Y. Cao C. Xie J. Ni J. Guan G., 2014 N-acetylglucosamine-induced white-to-opaque switching in *Candida albicans* is independent of the Wor2 transcription factor. Fungal Genet. Biol. 62: 71–77. 24161730 10.1016/j.fgb.2013.10.005

[bib73] Ucar D. Beyer A. Parthasarathy S. Workman C. T., 2009 Predicting functionality of protein-DNA interactions by integrating diverse evidence. Bioinformatics 25: i137–i144. 19477979 10.1093/bioinformatics/btp213 PMC2687967

[bib74] Vernes S. C. Oliver P. L. Spiteri E. Lockstone H. E. Puliyadi R., 2011 Foxp2 regulates gene networks implicated in neurite outgrowth in the developing brain. PLoS Genet. 7: e1002145. 21765815 10.1371/journal.pgen.1002145 PMC3131290

[bib75] Vinces M. D. Kumamoto C. A., 2007 The morphogenetic regulator Czf1p is a DNA-binding protein that regulates white–opaque switching in *Candida albicans*. Microbiology 153: 2877–2884. 17768232 10.1099/mic.0.2007/005983-0

[bib76] Wang H. Song W. Huang G. Zhou Z. Ding Y., 2011 *Candida albicans* Zcf37, a zinc finger protein, is required for stabilization of the white state. FEBS Lett. 585: 797–802. 21315072 10.1016/j.febslet.2011.02.005

[bib77] Wei Y. Wu G. Ji H., 2013 Global mapping of transcription factor binding sites by sequencing chromatin surrogates: a perspective on experimental design, data analysis, and open problems. Stat. Biosci. 5: 156–178. 23762209 10.1007/s12561-012-9066-5 PMC3677239

[bib78] Whitfield T. W. Wang J. Collins P. J. Partridge E. C. Aldred S. F., 2012 Functional analysis of transcription factor binding sites in human promoters. Genome Biol. 13: R50. 22951020 10.1186/gb-2012-13-9-r50 PMC3491394

[bib79] Wu G. Ji H., 2013 ChIPXpress: using publicly available gene expression data to improve ChIP-seq and ChIP-chip target gene ranking. BMC Bioinformatics 14: 188. 23758851 10.1186/1471-2105-14-188 PMC3684512

[bib80] Wu J. Smith L. T. Plass C. Huang T. H., 2006 ChIP-chip comes of age for genome-wide functional analysis. Cancer Res. 66: 6899–6902. 16849531 10.1158/0008-5472.CAN-06-0276

[bib81] Xu N. Dong Y. J. Yu Q. L. Zhang B. Zhang M., 2015 Convergent regulation of *Candida albicans* Aft2 and Czf1 in invasive and opaque filamentation. J. Cell. Biochem. 116: 1908–1918. 25716417 10.1002/jcb.25146

[bib82] Yi S. Sahni N. Daniels K. J. Pujol C. Srikantha T., 2008 The same receptor, G protein, and mitogen-activated protein kinase pathway activate different downstream regulators in the alternative white and opaque pheromone responses of *Candida albicans*. Mol. Biol. Cell 19: 957–970. 18162580 10.1091/mbc.E07-07-0688 PMC2262975

[bib83] Yi S. Sahni N. Daniels K. J. Lu K. L. Huang G., 2011 a Self-induction of **a/a** or α/α biofilms in *Candida albicans* is a pheromone-based paracrine system requiring switching. Eukaryot. Cell 10: 753–760. 21498642 10.1128/EC.05055-11 PMC3127667

[bib84] Yi S. Sahni N. Daniels K. J. Lu K. L. Srikantha T., 2011 b Alternative mating type configurations (**a**/α *vs.* **a**/**a** or α/α) of *Candida albicans* result in alternative biofilms regulated by different pathways. PLoS Biol. 9: e1001117. 21829325 10.1371/journal.pbio.1001117 PMC3149048

[bib85] Yu X. Li L. Zola J. Aluru M. Ye H., 2011 A brassinosteroid transcriptional network revealed by genome‐wide identification of *BESI* target genes in *Arabidopsis thaliana*. Plant J. 65: 634–646. 21214652 10.1111/j.1365-313X.2010.04449.x

[bib86] Zordan R. E. Galgoczy D. J. Johnson A. D., 2006 Epigenetic properties of white–opaque switching in *Candida albicans* are based on a self-sustaining transcriptional feedback loop. Proc. Natl. Acad. Sci. USA 103: 12807–12812. 16899543 10.1073/pnas.0605138103 PMC1535343

[bib87] Zordan R. E. Miller M. G. Galgoczy D. J. Tuch B. B. Johnson A. D., 2007 Interlocking transcriptional feedback loops control white-opaque switching in *Candida albicans*. PLoS Biol. 5: e256. 17880264 10.1371/journal.pbio.0050256 PMC1976629

