# Supplementary material for: Binding Sites in the EFG1 Promoter for Transcription Factors in a Proposed Regulatory Network: A Functional Analysis in the White and Opaque Phases of Candida albicans
Source: G3 (Bethesda). 2016 Apr 20;6(6):1725–37. doi: 10.1534/g3.116.029785 (PMC4889668; doi:10.1534/g3.116.029785)
Supplement: Supplemental Material [file supp_6_6_1725__index.html]

Binding Sites in the EFG1 Promoter for Transcription Factors in a Proposed Regulatory Network: A Functional Analysis in the White and Opaque Phases of Candida albicans — Supplemental Material 

# Binding Sites in the *EFG1* Promoter for Transcription Factors in a Proposed Regulatory Network: A Functional Analysis in the White and Opaque Phases of *Candida albicans*

## Supplemental Material for Pujol *et al.*, 2016

**Files in this Data Supplement:**

- Figure S1 - Comparisons of the strategies used by Lachke et al. (2003) (A) and the present study (B). (.pdf, 193 KB)
- Figure S2 - A diagram for the strategy for inserting *RLUC* into one copy of the *EFG1* locus. (.pdf, 220 KB)
- Table S1 - Strains used. (.pdf, 366 KB)
- Table S2 - Primers used. (.pdf, 234 KB)
- Table S3 - Deletion cassettes used to generate promoter derivatives. (.pdf, 214 KB)
