# Supplementary material for: Binding Sites in the EFG1 Promoter for Transcription Factors in a Proposed Regulatory Network: A Functional Analysis in the White and Opaque Phases of Candida albicans
Source: G3 (Bethesda). 2016 Apr 20;6(6):1725–37. doi: 10.1534/g3.116.029785 (PMC4889668; doi:10.1534/g3.116.029785)

**Figure S1.** Comparisons of the strategies used by Lachke et al. (2003) (A) and the present study (B).

A. *EFG1* locus configuration in Lachke et al. (2003) study.

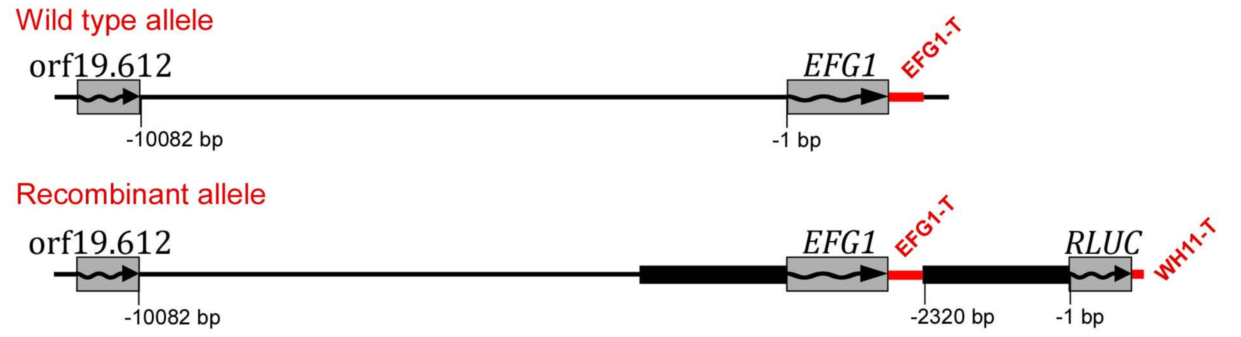


B. *EFG1* locus configuration in the present study. *EFG1* orf replaced with *RLUC* downstream of *EFG1* promoter region.

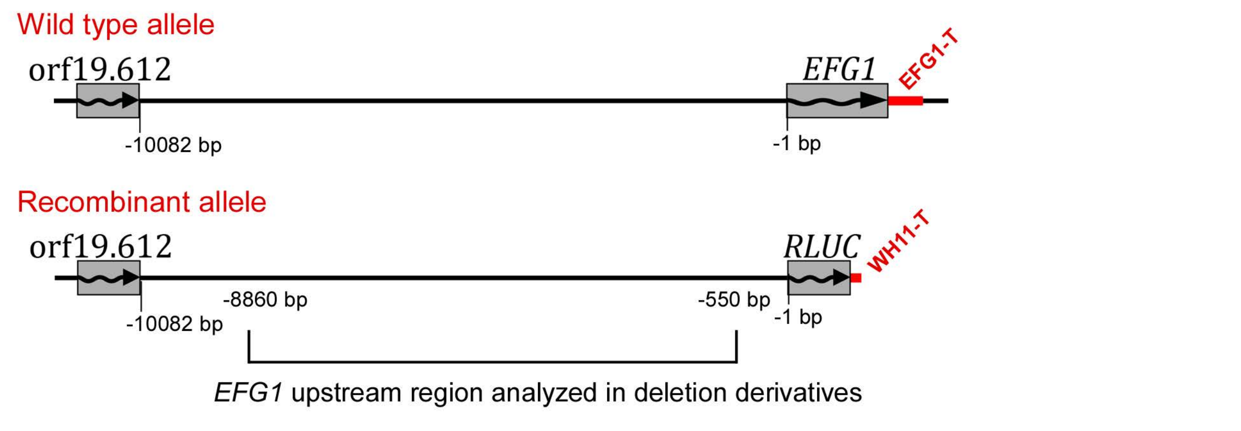

Supplement: Supplemental Material [file supp_g3.116.029785_FigureS1.pdf]
