# Supplementary material for: Binding Sites in the EFG1 Promoter for Transcription Factors in a Proposed Regulatory Network: A Functional Analysis in the White and Opaque Phases of Candida albicans
Source: G3 (Bethesda). 2016 Apr 20;6(6):1725–37. doi: 10.1534/g3.116.029785 (PMC4889668; doi:10.1534/g3.116.029785)

Figure S2. A diagram of the strategy for inserting *RLUC* into one copy of the *EFG1* locus. WH11-ter, *WH11* termination sequence. ACT-ter, actin termination sequence. Orf19.612, the first gene upstream of *EFG1*. A. Insertion diagram. B. Configuration of deletion derivatives.

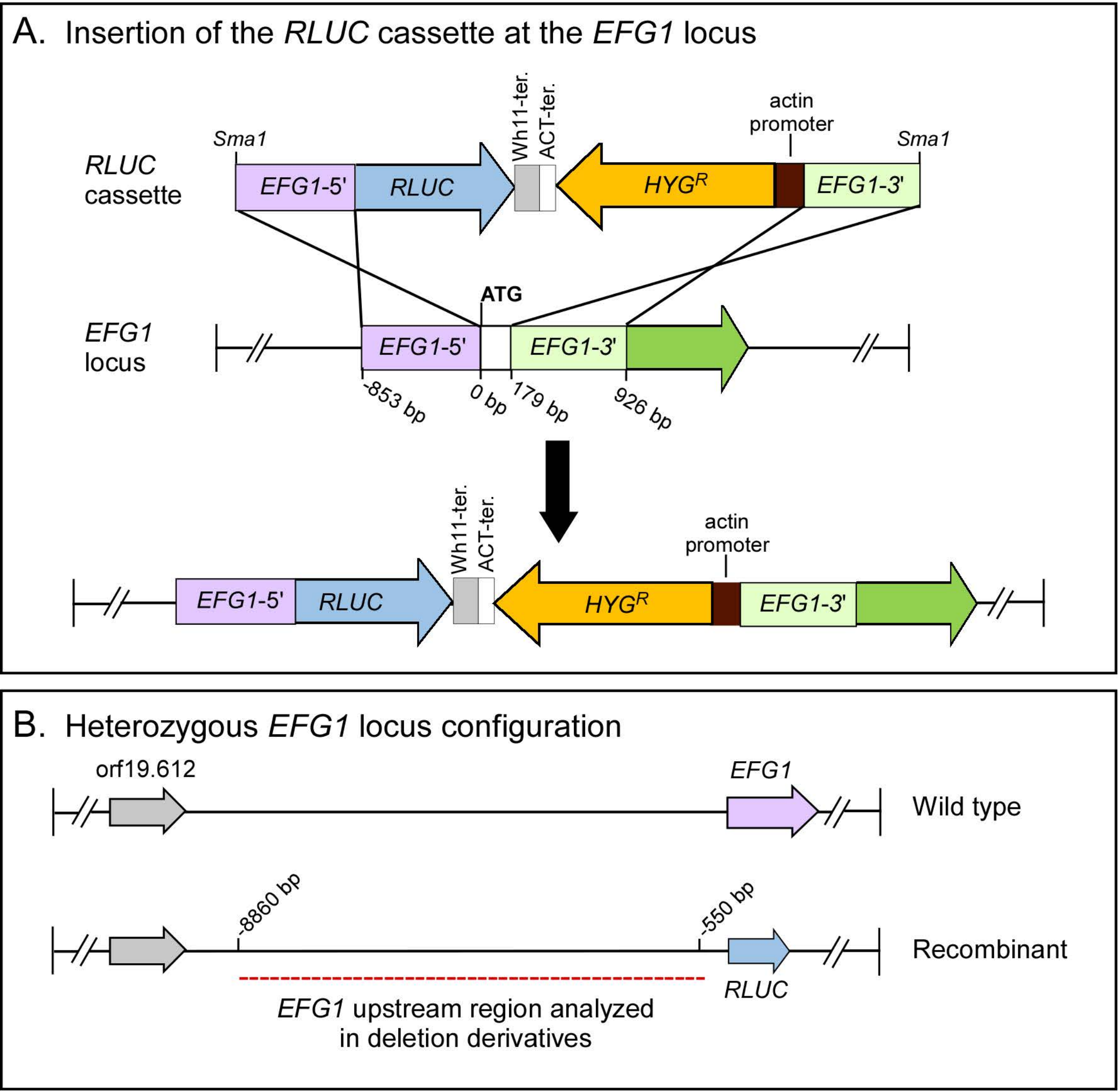

Supplement: Supplemental Material [file supp_g3.116.029785_FigureS2.pdf]
