# Supplementary material for: Binding Sites in the EFG1 Promoter for Transcription Factors in a Proposed Regulatory Network: A Functional Analysis in the White and Opaque Phases of Candida albicans
Source: G3 (Bethesda). 2016 Apr 20;6(6):1725–37. doi: 10.1534/g3.116.029785 (PMC4889668; doi:10.1534/g3.116.029785)
Supplement: Supplemental Material [file supp_g3.116.029785_TableS1.pdf]

Table S1. Strains used

Strain	Parent strain	Genotype[a]	Promoter derivative	Source
WO-1 (α/α)		Blood stream isolate/IA (USA)	—	[1]
F1	WO-1	*EFG1/efg1::RLUC-HYG^R*	WT	This study
ΔP1-2	F1	*EFG1/[p-efg1Δ(-8856 to -8054)::FRT]efg1::RLUC-HYG^R*	ΔP1	This study
ΔP1-3	F1	*EFG1/[p-efg1Δ(-8856 to -8054)::FRT]efg1::RLUC-HYG^R*		This study
ΔP2-2	F1	*EFG1/[p-efg1Δ(-7828 to -6867)::FRT]efg1::RLUC-HYG^R*	ΔP2	This study
ΔP2-3	F1	*EFG1/[p-efg1Δ(-7828 to -6867)::FRT]efg1::RLUC-HYG^R*		This study
ΔP3-2	F1	*EFG1/[p-efg1Δ(-6461 to -5897)::FRT]efg1::RLUC-HYG^R*	ΔP3	This study
ΔP3-3	F1	*EFG1/[p-efg1Δ(-6461 to -5897)::FRT]efg1::RLUC-HYG^R*		This study
ΔP4-2	F1	*EFG1/[p-efg1Δ(-5884 to -5209)::FRT]efg1::RLUC-HYG^R*	ΔP4	This study
ΔP4-3	F1	*EFG1/[p-efg1Δ(-5884 to -5209)::FRT]efg1::RLUC-HYG^R*		This study
ΔP5-1	F1	*EFG1/[p-efg1Δ(-5196 to -4303)::FRT]efg1::RLUC-HYG^R*	ΔP5	This study
ΔP5-3	F1	*EFG1/[p-efg1Δ(-5196 to -4303)::FRT]efg1::RLUC-HYG^R*		This study
ΔP6-2	F1	*EFG1/[p-efg1Δ(-4366 to -3492)::FRT]efg1::RLUC-HYG^R*	ΔP6	This study
ΔP6-3	F1	*EFG1/[p-efg1Δ(-4366 to -3492)::FRT]efg1::RLUC-HYG^R*		This study
ΔP7-1	F1	*EFG1/[p-efg1Δ(-3493 to -2774)::FRT]efg1::RLUC-HYG^R*	ΔP7	This study
ΔP7-2	F1	*EFG1/[p-efg1Δ(-3493 to -2774)::FRT]efg1::RLUC-HYG^R*		This study
ΔP8-2	F1	*EFG1/[p-efg1Δ(-2836 to -2250)::FRT]efg1::RLUC-HYG^R*	ΔP8	This study
ΔP8-4	F1	*EFG1/[p-efg1Δ(-2836 to -2250)::FRT]efg1::RLUC-HYG^R*		This study
ΔP9-4	F1	*EFG1/[p-efg1Δ(-2218 to -1693)::FRT]efg1::RLUC-HYG^R*		This study

ΔP9-5	F1	*EFG1/[p-efg1Δ(-2218 to -1693)::FRT]efg1::RLUC-HYG*^{R}	ΔP9	This study
ΔP10-1	F1	*EFG1/[p-efg1Δ(-1181 to -555)::FRT]efg1::RLUC-HYG*^{R}	ΔP10	This study
ΔP10-3	F1	*EFG1/[p-efg1Δ(-1181 to -555)::FRT]efg1::RLUC-HYG*^{R}		This study
ΔP1-8-1	F1	*EFG1/[p-efg1Δ(-8856 to -2250)::FRT]efg1::RLUC-HYG*^{R}	ΔP1-8	This study
ΔP1-8-2	F1	*EFG1/[p-efg1Δ(-8856 to -2250)::FRT]efg1::RLUC-HYG*^{R}		This study
ΔP1-9-1	F1	*EFG1/[p-efg1Δ(-8856 to -1693)::FRT]efg1::RLUC-HYG*^{R}	ΔP1-9	This study
ΔP1-9-2	F1	*EFG1/[p-efg1Δ(-8856 to -1693)::FRT]efg1::RLUC-HYG*^{R}		This study
ΔP1- WhTSP-1	F1	*EFG1/[p-efg1Δ(-8856 to -1015)::FRT]efg1::RLUC-HYG*^{R}	ΔP1- WhTSP	This study
ΔP1- WhTSP-2	F1	*EFG1/[p-efg1Δ(-8856 to -1015)::FRT]efg1::RLUC-HYG*^{R}		This study
ΔP2,P3-1	ΔP2-2	*EFG1/[p-efg1Δ(-7828 to -6867)::FRT, Δ(-6461 to -5897)::FRT]efg1::RLUC-HYG*^{R}	ΔP2,P3	This study
ΔP2,P3-4	ΔP2-3	*EFG1/[p-efg1Δ(-7828 to -6867)::FRT, Δ(-6461 to -5897)::FRT]efg1::RLUC-HYG*^{R}		This study
ΔP1,P2,P3-2	ΔP2,P3 -1	*EFG1/[p-efg1Δ(-8856 to -8054)::FRT, Δ(-7828 to -6867)::FRT, Δ(-6461 to -5897)::FRT]efg1::RLUC-HYG*^{R}	ΔP1,P2,P3	This study
ΔP1,P2,P3-3	ΔP2,P3 -4	*EFG1/[p-efg1Δ(-8856 to -8054)::FRT, Δ(-7828 to -6867)::FRT, Δ(-6461 to -5897)::FRT]efg1::RLUC-HYG*^{R}		This study
ΔP9,P10-3	ΔP10-1	*EFG1/[p-efg1Δ(-2218 to -1693)::FRT, Δ(-1181 to -555)::FRT]efg1::RLUC-HYG*^{R}	ΔP9,P10	This study
ΔP9,P10-6	ΔP10-3	*EFG1/[p-efg1Δ(-2218 to -1693)::FRT, Δ(-1181 to -555)::FRT]efg1::RLUC-HYG*^{R}		This study

P37005 (**a**/**a**)		*Healthy (oral)/FL (USA)*	—	[2]
I1	P37005	*EFG1/efg1::RLUC-HYG^R*	WT	This study
PΔP1-1	I1	*EFG1/[p-efg1Δ(-8856 to -8054)::FRT]efg1::RLUC-HYG^R*	ΔP1	This study
PΔP1-2	I1	*EFG1/[p-efg1Δ(-8856 to -8054)::FRT]efg1::RLUC-HYG^R*		This study
PΔP2-1	I1	*EFG1/[p-efg1Δ(-7828 to -6867)::FRT]efg1::RLUC-HYG^R*	ΔP2	This study
PΔP2-3	I1	*EFG1/[p-efg1Δ(-7828 to -6867)::FRT]efg1::RLUC-HYG^R*		This study
PΔP4-1	I1	*EFG1/[p-efg1Δ(-5884 to -5209)::FRT]efg1::RLUC-HYG^R*	ΔP4	This study
PΔP4-2	I1	*EFG1/[p-efg1Δ(-5884 to -5209)::FRT]efg1::RLUC-HYG^R*		This study
PΔP9-3	I1	*EFG1/[p-efg1Δ(-2218 to -1693)::FRT]efg1::RLUC-HYG^R*	ΔP9	This study
PΔP9-6	I1	*EFG1/[p-efg1Δ(-2218 to -1693)::FRT]efg1::RLUC-HYG^R*		This study
PΔP10-1	I1	*EFG1/[p-efg1Δ(-1181 to -555)::FRT]efg1::RLUC-HYG^R*	ΔP10	This study
PΔP10-2	I1	*EFG1/[p-efg1Δ(-1181 to -555)::FRT]efg1::RLUC-HYG^R*		This study
PΔP1-9-1	I1	*EFG1/[p-efg1Δ(-8856 to -1693)::FRT]efg1::RLUC-HYG^R*	ΔP1-9	This study
PΔP1-9-3	I1	*EFG1/[p-efg1Δ(-8856 to -1693)::FRT]efg1::RLUC-HYG^R*		This study

[a]. *p-efg1Δ(-X to -Y)*: deletion of the *EFG1* promoter from position -X to position -Y, in bp, relative to the *EFG1* start codon.

References

1. Slutsky B, Staebell M, Anderson J, Risen L, Pfaller M, and Soll DR. (1987) “White-Opaque transition”: a second High-frequencyswitching system in *Candida albicans*. Journal of Bacteriology 169: 189-197.

2. Lockhart SR, Pujol C, Daniels KJ, Miller MG, Johnson AD, Pfaller M, and Soll DR. (2002) In *Candida albicans*, white-opaque switchers are homozygous for mating type. Genetics 162: 737-745.
