# Supplementary material for: Binding Sites in the EFG1 Promoter for Transcription Factors in a Proposed Regulatory Network: A Functional Analysis in the White and Opaque Phases of Candida albicans
Source: G3 (Bethesda). 2016 Apr 20;6(6):1725–37. doi: 10.1534/g3.116.029785 (PMC4889668; doi:10.1534/g3.116.029785)
Supplement: Supplemental Material [file supp_g3.116.029785_TableS2.pdf]

Table S2. Primers used

Primer name	Sequence	Purpose
efg5primef1	TCACCCGGGGCATTTAGCTGCTATTTCAACC	EFG1-5’
efg5primer1.2	TCACCTGCAGGTAATATGGGTTATATTCTTGGT	EFG1-5’
efg3primef2.2	TCACCTGCAGGTCCAACAGCCTTATAACTAC	EFG1-3’
efg3primer2	TCACCCGGGATCACTCGTTTAATATCTCTGAC	EFG1-3’
CaHYgB-1	ATATGTCGACATGAAAAAACCAGAATTGACTGC	*HYG*^{R} from pBSIICaHygB
CaHygB-2	TATACATATGTTATTCTTTAGCTCTTGGTCTAG	*HYG*^{R} from pBSIICaHygB
ACT1P-1	TATACTCGAGTTTGAATGATTATATTTTTTTAATATT	*ACT1* promoter
ACT1P-2	CCATCATAAAATCTCGAGCGTCAAAAC	*ACT1* promoter
hynancof-2a	TCATCATGAGCGTCAAAACTAGAGAATAAT	*HYG*^{R} from pCaHygB
hynaw3ncor-2	TCATCATGACCTGCAGGTAAATAGTAATAATTACCA	*HYG*^{R} from pCaHygB
rlucsbff-2	TCACCTGCAGGAGCTTAAAGATGACTTCGAAAGTTTAT	*HYG*^{R} + *RLUC*
efg5’chk	CATCCTGTTTAAAGTCTTTCC	Integration check for *RLUC* cassette
rlucrchk2	CATCTGGCCCACCACTGCGG	Integration check for *RLUC* cassette
pEFG1Δ5'F	ACGATGGCAACGTTGGATTTTACC	5’ for ΔP1, ΔP1-8, ΔP9, and ΔP1-WhTSP cassettes
pEFG1Δ5'R	AATTCCCGGGTTATCATCGTCGTTTGGTGAGA	5’ for ΔP1, ΔP1-8, ΔP9, and ΔP1-WhTSP cassettes
ΔP1-3’F	GAGTCCCGGGTTTACAAAGATAG	3’ for ΔP1 cassette
ΔP1-3’R	CAACACTGAAGGCTAGAACT	3’ for ΔP1 cassette

ΔP2-5'F	TCTCACCAAACGACGATGAT	5' for ΔP2 cassette
ΔP2-5'R	TCCTCCCGGGTGTTTAGCAAGCTTCGTA	5' for ΔP2 cassette
ΔP2-3'F	TTATCCCGGGTAAGCAAAAACAAACACGACA	3' for ΔP2 cassette
ΔP2-3'R	CCATTCACTTTAGTCTCTCG	3' for ΔP2 cassette
ΔP3-5'F	CACAGCATGTTTCAGAAGAG	5' for ΔP3 cassette
ΔP3-5'R	CATTCCCGGGTGTCTACATTTGTAACG	5' for ΔP3 cassette
ΔP3-3'F	AAAACCCGGGAGACTAAAGTGAATGGC	3' for ΔP3 cassette
ΔP3-3'R	CAACTCTACTAGCATTAACG	3' for ΔP3 cassette
ΔP4,5-5'F	AAGGATTGTTGACGCAAAGA	5' for ΔP4 and ΔP5 cassettes
ΔP4-5'R	ACTTCCCGGGCACTTTAGTCTCTCGTTATTTGAC	5' for ΔP4 cassette
ΔP4-3'F	TTATCCCGGGTTTCATGCGATTACGGCA	3' for ΔP4 cassette
ΔP4-3'R	GACCAAAGCCCTCAGTATTG	3' for ΔP4 cassette
ΔP5-5'R	ATTACCCGGGATCGCATGAAACGCGGCATA	5'for ΔP5 cassette
ΔP5-3'F	ATAACCCGGGAATAAGAAAAGGCAGA	3' for ΔP5 cassette
ΔP5-3'R	GTAAAACACCACTGAACACA	3' for ΔP5 cassette
ΔP6-5'F	ATGTTGTTTTCCTTATGCCG	5' for ΔP6 cassette
ΔP6-5'R	GGTTCCCGGGTGTTGTTGTAAGAGTAGG	5' for ΔP6 cassette
ΔP6-3'F	GGTGCCCGGGTGAGTTGGTGTATTCT	3' for ΔP6 cassette
ΔP6-3'R	CTCACAAGGGTCAAGGTTAT	3' for ΔP6 cassette
ΔP7-5'F	AACAAAAACCTAGCAACGGG	5' for ΔP7 cassette
ΔP7-5'R	CTCACCCGGGCACCCATCACAAATCAC	5' for ΔP7 cassette
ΔP7-3'F	AACTCCCGGGTCTATTGTCTGTCTTGATGG	3' for ΔP7 cassette
ΔP7-3'R	ACACTACCGCTTTTGTAACT	3' for ΔP7 cassette
ΔP8-5'F	GGCTTTGGTCGTGGTTCTAA	5' for ΔP8 cassette
ΔP8-5'R	CCTTCCCGGGTCGAAAAAATTGGTTGTTCACG	5' for ΔP8 cassette

ΔP8-3’F	TTGT<u>CCCGGG</u>TTTACAACACTGAGGTGTCAA	3’ for ΔP8 and ΔP1-8 cassettes
ΔP8-3’R	CCTGTTGGTAGTATGGAATA	3’ for ΔP8 and ΔP1-8 cassettes
ΔP9-5’F	GTAGGTTTGTGTTCAGTGGT	5’ for ΔP9 and ΔP10 cassettes
ΔP9-5’R	TTGT<u>CCCGGG</u>TTCAATTTCTTGACACCTCAG	5’ for ΔP9 cassette
ΔP9-3’F	TATA<u>CCCGGG</u>AGCAATACTGCACAATAACAAC	3’ for ΔP9 and ΔP1-9 cassettes
ΔP9-3’R	TAAAGTTGGTGTTCGGGTTA	3’ for ΔP9 and ΔP1-9 cassettes
ΔP10-5’R	TTTT<u>CCCGGG</u>GCAAAGGTTTGGATCCTTTTGG	5’ for ΔP10 cassette
ΔP10-3’F	TACC<u>CCCGGG</u>AAGCTATATATTTGATTTAGTG	3’ for ΔP10 cassette
ΔP10-3’R	CTTGGTAGTCAAATAGAACAAATC	3’ for ΔP10 cassette
ΔWhTSP-3’F	TCAT<u>CCCGGG</u>TAAAGTCTTTCCGAGTAATT	3’ for ΔP1-WhTSP cassette
ΔWhTSP-3’R	ACGTGACCTGAGCAACTAAT	3’ for ΔP1-WhTSP cassette
SATtestB	CAGCGATGTACTGGTACTGGT	Integration check for deletion cassettes
notSAT	GAAATCCAGACAGTCGAGTTAGACA	Integration check for deletion cassettes
rlucRchk	ACAGCATTTTCTGCATGT	Integration check for deletion cassettes
