# Supplementary material for: Binding Sites in the EFG1 Promoter for Transcription Factors in a Proposed Regulatory Network: A Functional Analysis in the White and Opaque Phases of Candida albicans
Source: G3 (Bethesda). 2016 Apr 20;6(6):1725–37. doi: 10.1534/g3.116.029785 (PMC4889668; doi:10.1534/g3.116.029785)
Supplement: Supplemental Material [file supp_g3.116.029785_TableS3.pdf]

Table S3. Deletion cassettes used to generate promoter derivatives

Cassette	Deleted region	
	Position relative to the *EFG1* start codon (bp)	Size (bp)
ΔP1	-8856 to -8054	803
ΔP2	-7828 to -6867	962
ΔP3	-6461 to -5897	565
ΔP4	-5884 to -5209	676
ΔP5	-5196 to -4303	894
ΔP6	-4366 to -3492	875
ΔP7	-3493 to -2774	720
ΔP8	-2836 to -2250	577
ΔP9	-2218 to -1693	526
ΔP10	-1181 to -555	627
ΔP1-8	-8856 to -2250	6597
ΔP1-9	-8856 to -1693	7164
ΔP1-WhTSP	-8856 to -1015	7842

The position of the deleted regions correspond to those found at the *Candida* Genome Database (http://www.candidagenome.org) for the *Candida albicans* SC5314 assembly 22 of chromosome 1A.
